# Single-cell RNA-sequencing uncovers compound kushen injection synergistically improves the efficacy of chemotherapy by modulating the tumor environment of breast cancer

**DOI:** 10.3389/fimmu.2022.965342

**Published:** 2022-10-31

**Authors:** Xinkui Liu, Meirong Bai, Huiying Li, Peizhi Ye, Xiaoxia Duan, Chao Wu, Zhihong Huang, Shan Lu, Jingyuan Zhang, Zihan Zhao, Fengying Guo, Rongli You, Wenjie Qin, Wei Wang, Aiqing Han, Liangliang Shen, Yitao Wang, Zheng Zhao, Hua Luo, Jiarui Wu

**Affiliations:** ^1^ School of Chinese Pharmacy, Beijing University of Chinese Medicine, Beijing, China; ^2^ Key Laboratory of Mongolian Medicine Research and Development Engineering, Ministry of Education, Tongliao, China; ^3^ College of Biological Sciences and Technology, Beijing Key Laboratory of Food Processing and Safety in Forestry, Beijing Forestry University, Beijing, China; ^4^ National Cancer Center, National Clinical Research Center for Cancer, Chinese Medicine Department of the Cancer Hospital of the Chinese Academy of Medical Sciences and Peking Union Medical College, Beijing, China; ^5^ Beijing Zest Bridge Medical Technology Inc., Beijing, China; ^6^ School of Management, Beijing University of Chinese Medicine, Beijing, China; ^7^ Beijing Zhendong Pharmaceutical Research Institute Co., Ltd., Beijing, China; ^8^ Macau Centre for Research and Development in Chinese Medicine, State Key Laboratory of Quality Research in Chinese Medicine, Institute of Chinese Medical Sciences, University of Macau, Macao, Macao SAR, China

**Keywords:** compound kushen injection, triple-negative breast cancer, 4T1 tumor bearing mice, single cell RNA-sequencing, T lymphocytes

## Abstract

**Background:**

Due to lack of enough specific targets and the immunosuppressive tumor microenvironment (TME) of triple-negative breast cancer (TNBC), TNBC patients often cannot benefit from a single treatment option. This study aims to explore the regulatory effects of Compound kushen injection (CKI) plus chemotherapy on the TME of TNBC from a single cell level.

**Methods:**

A mouse TNBC model in BALB/c mice was established to evaluate the antitumor efficacy and toxicity of CKI combined with chemotherapy. Flow cytometry was used to observe the influence of CKI on the lymphocyte populations in the tumor bearing mice. Both bulk RNA sequencing (RNA-seq) and single-cell RNA-seq (scRNA-seq) were applied to portray the modulation of CKI combined with chemotherapy on the TME of TNBC mice.

**Results:**

CKI significantly enhanced the anticancer activity of chemotherapy *in vivo* with no obvious side effects. Flow cytometry results revealed a significantly higher activation of CD8^+^ T lymphocytes in the spleens and tumors of the mice with combination therapy. Bulk RNA-seq indicated that CKI could promote the cytotoxic immune cell infiltrating into tumor tissues. Meanwhile, scRNA-seq further revealed that CKI combined with chemotherapy could enhance the percentage of tumor-infiltrating CD8^+^ T cells, inhibit tumor-promoting signaling pathways, and promote T cell activation and positive regulation of immune response. In addition, CKI showed obvious anticancer activity against MDA-MB-231 breast tumor cells *in vitro*.

**Conclusions:**

The combination of CKI and chemotherapy might provide a higher efficiency and lower toxicity strategy than a single chemotherapy drug for TNBC. CKI potentiates the anti-TNBC effects of chemotherapy by activating anti-tumor immune response in mice.

## Introduction

Due to the lack of well-recognized therapeutic targets and enough treatment options, triple-negative breast cancer (TNBC) as a highly heterogeneous breast cancer subtype still has a high risk of early relapse and visceral metastasis, a shorter disease-free survival, and a dismal prognosis (1, 2). Although conventional chemotherapy is the standard treatment for TNBC and has improved clinical outcomes, many patients with TNBC show limited responses and often develop chemotherapy resistance and recurrence (3–5). In recent years, the treatment strategy based on molecular subtyping and genomic profiling is beginning to be applied to refractory metastatic TNBC (6). Although immunotherapy exhibited promising therapeutic effects for TNBC in combination with chemotherapy or molecularly targeted therapy, only a small portion of patients respond well to the novel therapy (6–9). Thus, considering the complexity of immunotherapy and their normally high treatment costs, it becomes more meaningful to develop newer combination therapies with broad-spectrum anticancer activity, high efficiency and low toxicity for TNBC based on existing chemotherapy drugs (10).

The tumor microenvironment (TME) plays a crucial role in the regulation of tumor progression, and the components of TME have been recognized as a biomarker for predicting patients’ prognosis and response to therapy across various solid tumors (11, 12). Especially in TNBC, increased tumor-infiltrating lymphocytes (TILs) are not only associated with favorable outcomes but also have the potential to serve as an indicator of a better response to chemotherapy, neoadjuvant chemotherapy, as well as anti-programmed cell death 1 (PD-1) or anti-programmed cell death 1 ligand 1 (PD-L1) immunotherapy (12–17). Transcriptomics research provides an important technical support for the in-depth exploration of tumor complexity and heterogeneity, and it also promotes the discovery of novel biomarkers and the development of potentially useful therapeutic strategies (11, 18–20). Recent technological advances in single-cell RNA-seq (scRNA-seq) have enabled a more detailed presentation of the fundamental properties of tumor-infiltrating immune cells, and a comprehensive single cell atlas of tumor-infiltrating T cell subpopulations across various cancer types has been constructed (21, 22). Recently, single-cell analysis has been used to deciphering the complex TME of TNBC patients receiving anti-PD-1 treatment (21, 23), while no single cell studies have been reported in dissecting the mechanisms of Chinese patent medicine on TME. Therefore, dissecting the effects and underlying regulatory mechanisms of Chinese patent medicine on the TME of TNBC at a single cell level is urgently needed.

Compound kushen injection (CKI, also known as Yanshu injection) is a National Medical Products Administration (NMPA)-approved anticancer Chinese patent medicine in China, which is extracted from the roots of two medicinal plants Kushen (*Radix Sophorae Flavescentis*) and Baituling (*Rhizoma Heterosmilacis*) through standardized Good Manufacturing Practice (GMP) (24, 25). A variety of anti-tumor active compounds in CKI like matrine and oxymatrine have been widely described (26, 27). CKI alone or it combined with chemotherapy or radiotherapy has been extensively adopted in clinical practice to treat liver cancer, gastric cancer, breast cancer, lung cancer, colorectal cancer, and other cancer types, suggesting that CKI possesses a broad spectrum of anti-cancer property (27–36). It is noteworthy that accumulating evidence suggests that CKI not only synergizes the efficacy and reduces the toxic side effects of chemotherapy or radiotherapy, but also improves patients’ quality of life and immune functions (28, 30, 31). CKI is the second most commonly used anticancer Chinese patent medicine, and the use rate of CKI was highest among the Chinese patent medicines used in 17 cancer types. In addition, CKI is also the second frequently used anticancer Chinese patent medicine in breast cancer (37). For breast cancer, according to the results of a meta-analysis, which comprised 16 randomized controlled trials (RCTs) enrolling a total of 1,315 patients, CKI in combination with chemotherapy indicates a better performance status and a reduced adverse drug reactions in the postoperative patients with breast cancer (31). Moreover, it has been reported that CKI can suppress human breast cancer stem-like cells by mediating the inactivation of the canonical Wnt/β-catenin pathway (29). Interestingly, a recent research aiming to discuss the roles of CKI on tumor immunity reveals that CKI alleviates the immunosuppressive effects of tumor-associated macrophages and afterwards relieves the its immune suppression on CD8^+^ T cells, which eventually improves the effects of low-dose sorafenib and reduces the adverse effects of chemotherapy (24). These clinical and experimental evidence suggested that CKI has great potential to serve as an adjuvant for anticancer immunotherapy. Our previous research also revealed possible immunotherapy biomarkers of CKI on TNBC by transcriptome data mining (38). Therefore, considering the immunoregulatory effects of CKI on the TME of HCC and its broad-spectrum anticancer activity, we aimed to study whether the combination of CKI and chemotherapy could increase the anti-tumor immunity in TNBC.

In this study, we aimed to assess the efficiency and toxicity of CKI combined with chemotherapy in controlling TNBC tumor growth and study the regulatory effects of the combination therapy on TME from a single cell level. A mouse breast cancer model in BALB/c mice with 4T1 cells was established to evaluate the antitumor efficacy of CKI combined with chemotherapy. Flow cytometry was used to observe the influence of CKI on the lymphocyte populations in the mice bearing 4T1 tumors. Single cell and bulk RNA sequencing were used to portray the modulation of CKI combined with chemotherapy on the TME of 4T1 tumor bearing mice.

## Materials and methods

### Ultra-high performance liquid chromatography-tandem mass spectrometry analysis

CKI (Batch No: 20200329), with total alkaloid concentration of 25 mg/mL, was gifted by Beijing Zhendong Pharmaceutical Research Institute Co., Ltd., China. Nine control compounds were used in this study, including matrine (HPLC: 98.7%), oxymatrine (HPLC: 92.9%), sophocarpine (HPLC: 99.84%), oxysophocarpine (HPLC: 93.1%), sophoridine (HPLC: 98.8%), N-methylcytisine (HPLC ≥ 98.0%), macrozamin (HPLC: 98.9%), trifolirhizin (HPLC ≥ 98.0%), and sophoranol (HPLC ≥ 95.0%).

LC-MS/MS was conducted to identify the components of CKI. The CKI was diluted 50-fold in 40% methanol solution and mixed well by vortex. The solution was centrifuged at 13,000 rpm for 15 minutes, and the obtained supernatant was used for LC-MS analysis. Meanwhile, every single standard compound was also diluted in 40% methanol solution and the concentration was 1 ug/mL. LC analysis was performed on a Thermo UHPLC vanquish system with a ACQUITY UPLC HSS T3 column (2.1 mm×100 mm, 1.8 µm) at 25°C, with water-formic acid (H_2_O-FA) (99.9:0.1, v/v) as mobile phase A and acetonitrile-formic acid (ACN-FA) (99.9:0.1, v/v) as mobile phase B. A gradient elution with the flow rate of 0.3 mL/min was executed as follows: 95% buffer A:5% buffer B for 0-17 min, 2% buffer A:98% buffer B for 17-17.2 min, and 95% buffer A:5% buffer B for 17.2-20 min. The MS analysis was performed on a Thermo Q-Exactive HFX system, with the electrospray ionization (ESI) source in both positive and negative ion modes. The spray voltages were set to 3.8 kV and -3.0 kV in positive and negative ion modes, respectively. The source gas parameters for sheath gas, aux gas and spare gas were set at 45, 20 and 0 units, respectively. The temperatures of capillary and probe heater were set at 320°C and 370°C, respectively. The scan range was set at m/z 90-1300.

### Cell culture and *in vitro* experiments

MDA-MB-231 human triple-negative breast cancer cells (Bena Culture Collection, China) were cultured in Dulbecco’s Modified Eagle’s Medium (DMEM) (Corning, USA) supplemented with 10% fetal bovine serum (FBS) (Corning, USA) and 1% penicillin/streptomycin (Gibco, USA). 4T1 murine triple-negative breast cancer cells (Shanghai Fuheng Biotechnology Co., Ltd., China) were cultured in RPMI-1640 medium (Corning, USA) supplemented with 10% FBS (Corning, USA) and 1% penicillin/streptomycin (Gibco, USA). All cells were cultured at 37°C in a humidified incubator with 5% CO_2_.

The MDA-MB-231 cell line was used to investigate the anti-cancer effect of CKI. MDA-MB-231 cells were plated in 96-well plates in DMEM supplemented with 10% FBS and 1% penicillin/streptomycin, at a density of 2×10^5^ cells/mL and maintained at 37°C in a 5% CO_2_ humidified incubator for 24 h. The cells were then treated with different concentrations of CKI (0.125, 0.25, 0.5, 1, 2, 4, 8 and 16 mg/mL) for 24, 48 and 72 h respectively. Cell survival was assessed using Cell Counting Kit-8 (CCK-8) (Dojindo, Japan) reagent. The optical density (OD) was detected at 450 nm using a microplate reader (Molecular Devices, USA). Based on the half-maximal inhibitory concentration (IC_50_) values and cellular state, the MDA-MB-231 cells with 2 mg/mL CKI treatment for 48 h were chosen for bulk RNA sequencing (RNA-seq). For clone formation assay, MDA-MB-231 cells (500 cells/well) were placed into 6-well plates and cultured for 10 days, and then the cells were treated with 0.375, 0.75 and 1.5 mg/mL CKI for 10 days, respectively. For wound-healing assay, MDA-MB-231 cells were treated with 0.375, 0.75 and 1.5 mg/mL CKI for 6, 12, 24 and 48 h, respectively.

### Animal models

BALB/c mice (female, 4-5 weeks old) were obtained from SPF (Beijing) Biotechnology Co., Ltd. (China) and fed in a pathogen-free vivarium under standard conditions. The principles and experimental protocols of animals used were approved by the Animal Care and Protection Committee of Beijing University of Chinese Medicine. The 4T1 tumor cell suspension was diluted in PBS and injected subcutaneously (0.1 mL, 1×10^6^ cells/mouse) into the left flank of each mouse. When the tumor size grew to around 60 mm^3^, the mice were randomly divided into six groups (n=8 in each group). The mice in the control group were injected intraperitoneally with saline every day for 17 days. The mice in the CKI group were injected intraperitoneally with CKI (4 mL/kg) every day for 17 days. The mice in the cisplatin (DDP) group were injected intraperitoneally with DDP (3 mg/kg) every three days for six times. The mice in the paclitaxel (PTX) group were injected intravenously with PTX (10 mg/kg) every three days for six times. The mice in the DDP+CKI combination therapy group were injected intraperitoneally with CKI (4 mL/kg per day) and DDP (3 mg/kg once every 3 days) for 17 days. The mice in the PTX+CKI combination therapy group were injected intraperitoneally with CKI (4 mL/kg per day) and were injected intravenously with PTX (10 mg/kg once every 3 days) for 17 days. Changes in tumor sizes were determined by measuring the tumor diameter in two dimensions with a caliper every 3 days. The tumor volumes (V) were calculated based on the formula: V=(width^2^×length)×0.52. The mice were sacrificed after the completion of the experiment. Tumor tissues and major organs (liver, heart, spleen, lung, and kidney) were excised, washed, fixed, and embedded into paraffin. The fixed samples were then sliced, subjected to the hematoxylin and eosin (H&E) staining and optical microscopy study. The apoptosis of tumor tissues was evaluated by terminal deoxynucleotidyl transferase dUTP nick end labeling (TUNEL) staining. The TUNEL assay was performed with the *In Situ* Cell Death Detection Kit (Roche, USA) to detect the apoptotic cells in frozen sections of tumor tissues.

### Flow cytometry analysis

For flow cytometry analysis of *in vivo* experiments, mouse tumors and spleens were quickly excised and then mechanically dissociated with scissors in sterile PBS. Splenocytes were filtered in 70 mM cell strainers. Tumor fragments were digested in 10% FBS (Thermo-Gibco), 0.5 mg/mL Collagenase from Clostridium histolyticum Type IV (Sigma-Aldrich), 0.15 mg/mL DNase I (Sigma-Aldrich) and RPMI 1640 (Thermo-Gibco) for 60 min at 37°C with rotation to promote dissociation. Single-cell suspensions were passed through a 70 μm cell strainer. Red blood cells in spleen samples were then lysed with RBC lysis buffer (BioLegend) for 10 min at room temperature, and lysis reactions were quenched by the addition of 20 mL PBS. Samples were centrifuged at 300 g for 5 min at 4°C. Cells were initially blocked with TruStain FcX™ (anti-mouse CD16/32 antibody, BioLegend) for 10 min at 4°C before staining with antibody panels. Then cells were stained with fluorescently labeled antibodies at a 1:100 dilution in Cell Staining Buffer (BioLegend) for 30 min at 4°C. Single-cell suspensions were stained with panel 1: PE-Cy7 anti-mouse CD45 (BioLegend), FITC anti-mouse CD3 (BioLegend), BV510 anti-mouse CD8a (BioLegend), and PerCP/Cy5.5 anti-mouse CD4 (BioLegend); panel 2: PerCP-Cy5.5 anti-mouse CD45 (BioLegend), FITC anti-mouse CD3 (BioLegend), and APC anti-mouse CD49b (BioLegend). All data was acquired by BD LSRFortessa flow cytometer (BD Bioscience) and analyzed with FlowJo software.

### Bulk RNA-seq and data analysis

The tumor tissues of three mice in each group were used for bulk RNA-seq. Furthermore, the MDA-MB-231 cells with or without 2 mg/mL CKI treatment for 48 h were also used for bulk RNA-seq (n=6 per group). RNA-seq analysis was performed according to standard procedures, including RNA quantification and qualification, library preparation for transcriptome sequencing, clustering, and sequencing. Sequencing of total RNA from mice tumor tissues and MDA-MB-231 cells after indicated treatments was accomplished by Shanghai Applied Protein Technology Co., Ltd., China.

The quality of raw reads was checked with FastQC (version 0.11.9, Babraham Bioinformatics), and the sequences containing low-quality bases or adapters were trimmed using Trim_galore (version 0.6.6, Babraham Bioinformatics). The index of the reference genome (mouse: GRCm39, human: GRCh38; Ensembl Release 104) was built using the STAR software (version 2.7.9a) (39), and subsequently the trimmed reads were further aligned to the STAR genome index. Meanwhile, resulting bam files were sorted using samtools (version 1.10) (40). Then, the expression values of read counts and transcripts per million (TPM) were calculated by RSEM (v1.3.3) (41). Gene sets developed by Bagaev et al. (11), Jimenez-Sanchez et al. (42), and Sun et al. (43) were used with single-sample gene set enrichment analysis (ssGSEA) (44) to provide enrichment scores for each of the immune signatures. Human genes were transferred into murine homologous genes by manually retrieving the GeneCards website (https://www.genecards.org/). The moderated t-test based on an empirical Bayesian approach (implemented in the limma package (45)) was used to determine differential signatures and the significance level cutoff was set at *P* < 0.05. Differential expression analysis was performed with the DESeq2 package (46), and fold changes (FCs) were shrunk with the ashr method (implemented in the “lfcShrink” function of DESeq2) (47). The significance threshold for differential gene expression screening was set as adjusted *P* < 0.05 and |log_2_FC| > 1.

### 10x genomics single cell RNA sequencing

The tumor tissues of the mice treated with saline, CKI, PTX and PTX+CKI were used to perform scRNA-seq (3 mice in each group). Fresh surgically resected tissue was washed with sterile PBS and minced with scissors. The tissue samples were immediately transferred to pre-cooled Tissue Storage Solution (Shanghai Biotechnology Corporation) and were shipped at 4°C. Tissue processing was completed within 48 h of collection. The tissues were transported in sterile culture dish with 10 mL 1×Dulbecco’s Phosphate-Buffered Saline (DPBS; Thermo Fisher) on ice to remove the residual tissue storage solution, then minced on ice. We used dissociation enzyme 0.1% Type I Collagenase (Sigma) dissolved in RPMI 1640 (Thermo Fisher) with 10% Fetal Bovine Serum (FBS; Thermo Fisher) to digest the tissues. The tissues were dissociated at 37°C with a shaking speed of 50 rpm for about 40 min. The dissociated cells were repeatedly collected at interval of 20 min to increase cell yield and viability. Cell suspensions were filtered using a 70 μm nylon cell strainer and red blood cells were removed by 1×Red Blood Cell Lysis Solution (Thermo Fisher). Dissociated cells were washed with 1X DPBS containing 2% FBS. The cells were stained with 0.4% Trypan blue (Thermo Fisher) to check the viability on Countess^®^ II Automated Cell Counter (Thermo Fisher).

We performed 3’ gene expression profiling on the single-cell suspension using the Chromium™ Single Cell 3’ Solution from 10x Genomics, following the manufacturer’s instructions. Reverse transcription, cDNA amplification and library preparation were performed using the Chromium Next GEM Single Cell 3’ GEM, Library & Gel Bead Kit v3.1 (10x Genomics) according to manufacturer’s instructions, with about 20,000 cells loaded onto a 10x Genomics cartridge for each sample. Cell-barcoded 3’ gene expression libraries were sequenced on an Illumina NovaSeq 6000 platform with 150 bp paired-end reads. The construction and sequencing of RNA libraries were performed at Shanghai Biotechnology Corporation, China.

The Cell Ranger toolkit (10x Genomics, version 6.1.2) was used to align reads to mouse reference genome (mm10, GENCODE vM23/Ensembl 98), assign cell barcodes, and generate the unique molecular identifier (UMI) matrices. The resulting count matrices showing the number of transcripts (UMIs) of each gene for a given cell were analyzed with R software (version 4.2.1) and the Seurat package (version 4.1.1) (48) in R, according to the standard pipeline with default parameters, unless mentioned otherwise. Scrublet (49) was utilized to each sequencing library to detect potential doublets, and they were removed when we performed clustering for T cell subgroups. The cells expressing less than 500 genes and the genes detected in less than 10 cells were preliminary filtered out from the raw UMI matrix. Then, the number of genes and UMI counts for each cell were further quantified, and the cells with the threshold of 500-5500 genes, 1500-40000 UMIs, fewer than 10% mitochondrial gene counts and fewer than 1% hemoglobin genes counts were kept to ensure that most of the cells with high quality were included for downstream analyses. The UMI count data was normalized with regularized negative binomial regression (50) after regressing for the percentages of mitochondrial genes and cell cycle. Individual Seurat objects were then integrated using the canonical correlation analysis (CCA) method (51), and top 3,000 most variable genes from each sample were combined for CCA vector identification. T-Distributed Stochastic Neighbor Embedding (t-SNE) dimensional reduction and clustering were performed based on the 20 most informative principal components (PCs). Clusters of cells were identified with the Louvain algorithm by employing the “FindClusters” function in Seurat, with a resolution of 0.8. DEGs were defined by a Wilcoxon Rank Sum test implemented in the “FindAllMarkers” function from Seurat. Clusters that were identified as T lymphocytes were extracted, and a second round of dimension reduction and clustering was performed on these subsets for further distinguishing T cell populations. The gene list used for dysfunctional T cells were adopted from Li et al. (52), and the cytotoxic gene list was used (including Gzma, Gzmb, Gzmg, Gzmk, Gzmm, Prf1, Fasl, Ifng, Tnf, Il2ra and Il2) (53).

The 50 hallmark gene sets described in the Molecular Signatures Database (MSigDB, version 7.4.1) (54), the 29 gene signatures developed by Bagaev et al. (11) and the T cell signatures introduced by Sun et al. (43) were used to perform ssGSEA, respectively. The moderated t-test based on an empirical Bayesian approach (implemented in the limma package) was applied to determine significantly changed pathways, and the significance cutoff was set at *P* < 0.05. GSEA analysis was performed based on immune system-related pathways deposited in Kyoto Encyclopedia of Genes and Genomes (KEGG) and immune-related biological processes deposited in Gene Ontology (GO) by employing the clusterProfiler package (55).

### Statistical analysis

Unless otherwise stated, independent experiments were run at least in triplicates (n=3). The results were expressed as means ± standard error of the mean (SEM). The Shapiro-Wilk test was used to check the normality of the variables before comparison. Two-tailed unpaired Student’s t-test was used for comparison of variables with normal distribution between two groups. One-way ANOVA was used for comparison of normal distributed variables among more than two groups. *P* < 0.05 was considered statistically significant. The *P* values were adjusted for multiple test correction with the Benjamini-Hochberg algorithm to reduce false positive rates. All statistical analyses were done using R (version 4.2.1).

## Results

### Identification of main constituents in CKI

We performed both positive and negative ion modes for UHPLC-Q-Exactive-MS analysis. The base peak intensity (BPI) chromatogram profile of CKI was shown in **Supplementary Figure 1**. In the positive ion mode, we identified 14 peaks and 15 compounds, eight of which were unambiguously confirmed by comparing the accurate masses and retention times with reference standards (**Supplementary Figure 1A**). In the negative ion mode, we identified 14 peaks and 11 compounds, one of which were unambiguously confirmed by comparing the accurate masses and retention times with reference standards (**Supplementary Figure 1B**). The 26 compounds identified in this study were shown in **Supplementary Table 1**. These compounds included eight alkaloids and derivatives (4^1^, 5, 8, 9, 10, 11^1^, 11^2^, 11^3^, 13), five organic acids and derivatives (3, 14, 19, 20, 21), five organic oxygen compounds (2, 4^2^, 6, 16, 17, 18), three benzenoids (7, 12, 25), two phenylpropanoids and polyketides (24, 28), one organic nitrogen compound (1), one lipids and lipid-like molecule (22), and one organoheterocyclic compound (26). Consistent with previous findings (35, 38, 56), we have also determined six alkaloids matrine, oxymatrine, N-methylcytisine, sophoridine, sophocarpine and oxysophocarpine, which have been known as the primary active ingredients of CKI.

### Antitumor effects of CKI combined with chemotherapy *in vivo*

**Figure 1:**
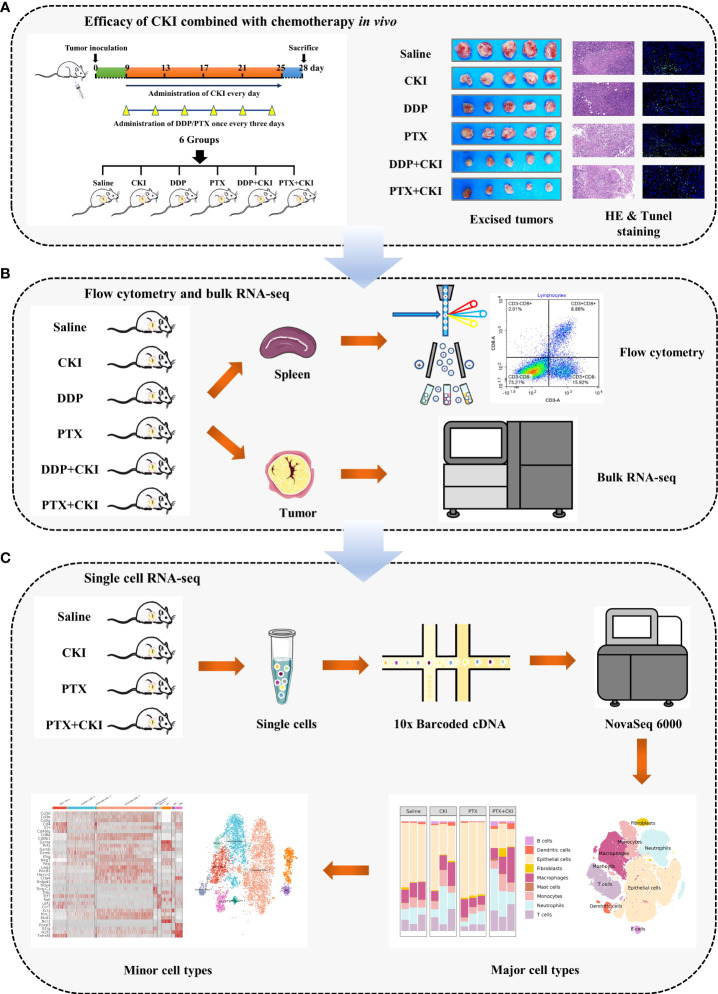
Workflow of this study. **(A)** Efficacy of DDP+CKI and PTX+CKI *in vivo*. **(B)** Flow cytometry and bulk RNA-seq analyses. **(C)** scRNA-seq analysis.

**Figure 2:**
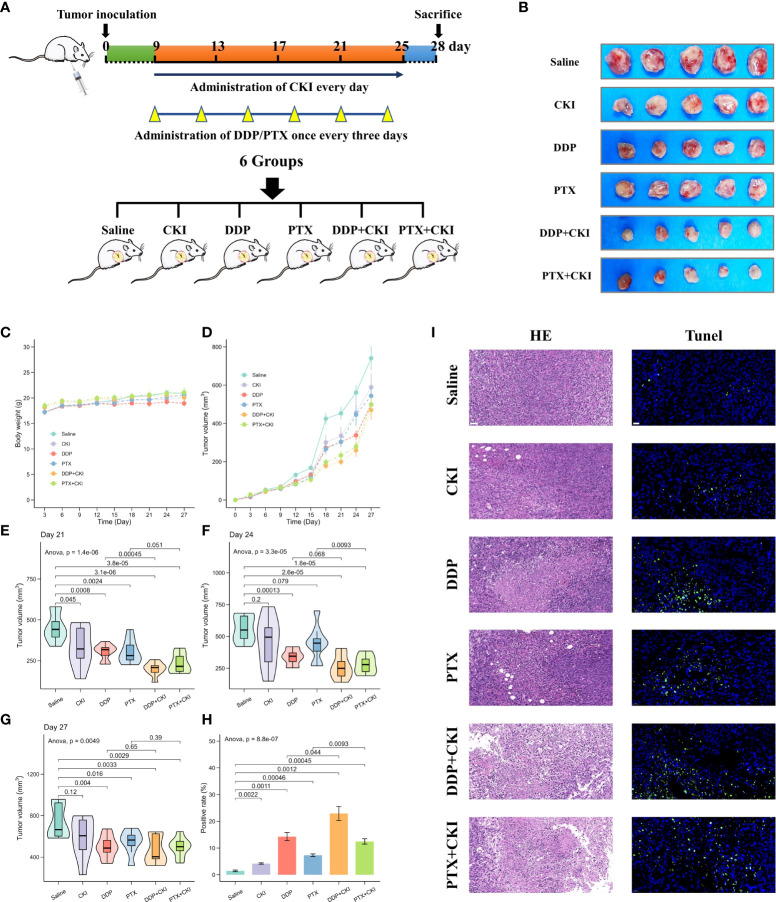
Efficacy of monotherapy and combination therapy on 4T1 mammary carcinomas. **(A)** Schematic representation of drug injection schedule. **(B)** Extracted tumor images of the mice from each treatment group on the last day after treatment. Only five samples in each treatment group were showed. **(C)** Body weight changes of mice burdened with 4T1 tumors in different groups. **(D)** Growth kinetics of 4T1 tumors in different groups. **(E)** Evaluation of 4T1 tumor volumes after treatment on day 21. **(F)** Evaluation of 4T1 tumor volumes after treatment on day 24. **(G)** Evaluation of 4T1 tumor volumes after treatment on day 27. **(H)** The apoptosis in tumor tissues quantified by counting the rate of TUNEL-positive cells. **(I)** Representative HE staining and TUNEL immunofluorescent staining images of tumor sections. For TUNEL staining, the green fluorescence represents apoptotic cells. The representative specimens were examined at 200x magnification for HE staining (scale bar=50 μm) and 400x magnification for TUNEL staining (scale bar=20 μm). Data represent the mean ± SEM (Figure 2C–G: n=8 in each group, Figure 2H: n=3 in each group).

**Figure 3:**
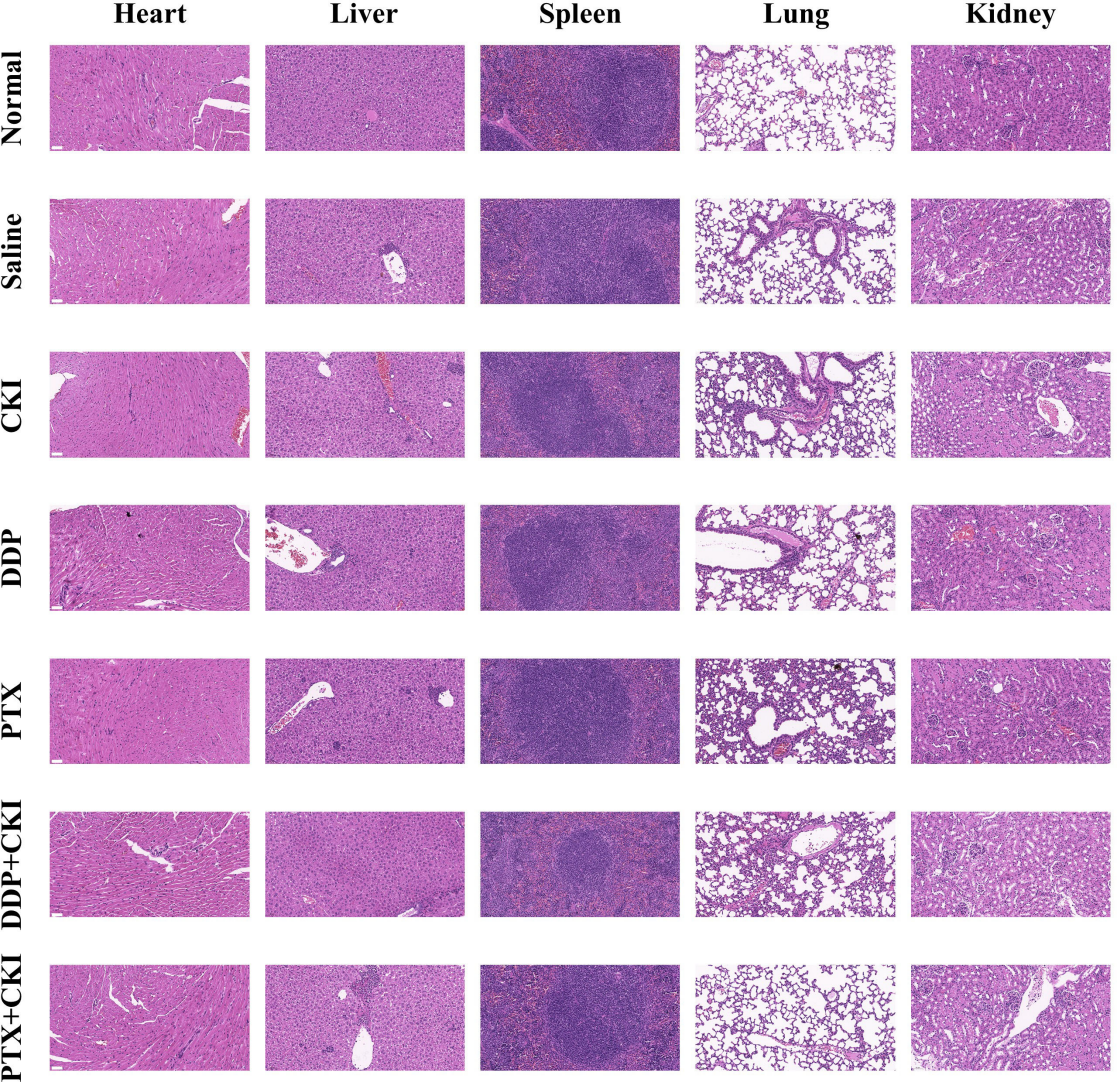
Representative HE staining images of major organs from mice treated with different formulations. The representative specimens were examined at 200x magnification (Scale bar=50 μm).

The overall workflow of *in vivo* experiments was shown in **Figure 1**. Antitumor efficacy of CKI, DDP, PTX, DDP+CKI, and PTX+CKI was assessed *in vivo* on a 4T1 tumor model (**Figure 2A**). Nine days after 4T1 transplantation in the abdominal subcutaneous tissue of female BALB/c mice, the mice bearing 4T1 tumors were treated with different therapeutic regimens to examine their antitumor efficacy. Two days after the last dose, mice were sacrificed and tumors were excised for further experiments (**Figure 2B**). Body weight of the mice did not change significantly in any of the groups during 27 days of administration (**Figure 2C**). Following the drug treatments, the tumor volumes of mice were measured (**Figures 2D–G**). Saline-treated mice (control) showed a faster growth in the breast tumor with time compared to all other groups (**Figure 2D**). The tumor volumes of DDP+CKI and PTX+CKI group were significantly smaller than that of the control group (**Figures 2E–G**). Compared to DDP and PTX monotherapy, the combination of CKI and DDP/PTX presented a more significant inhibitory effect on tumor growth (DDP+CKI vs. DDP on day 21, *P* < 0.001, **Figure 2E**; PTX+CKI vs. PTX on day 21, *P* = 0.051, **Figure 2E**; DDP+CKI vs. DDP on day 24, *P* = 0.068, **Figure 2E**; PTX+CKI vs. PTX on day 24, *P* < 0.01, **Figure 2F**). These results indicated that the anti-tumor effect of DDP and PTX *in vivo* could be effectively enhanced by CKI. Histopathological studies of isolated tumors of mice after drug treatment exhibited that all the five treatment groups promoted the apoptosis and necrosis of tumor cells, and the effect of combination therapy was superior to monotherapy (**Figure 2I** left). Meanwhile, the apoptosis of tumor tissues was detected by TUNEL staining. The results illustrated that the combination therapy group had much more apoptotic cells compared with all other groups (**Figure 2H**, **Figure 2I** right), which further confirmed that the combination of CKI with chemotherapy synergistically suppressed tumor growth. No noticeable abnormality was found in the heart, liver, spleen, lung, or kidney functions (**Figure 3**).

### The combination of CKI and chemotherapy enhances the proportion of immune cell subpopulations *in vivo*

**Figure 4:**
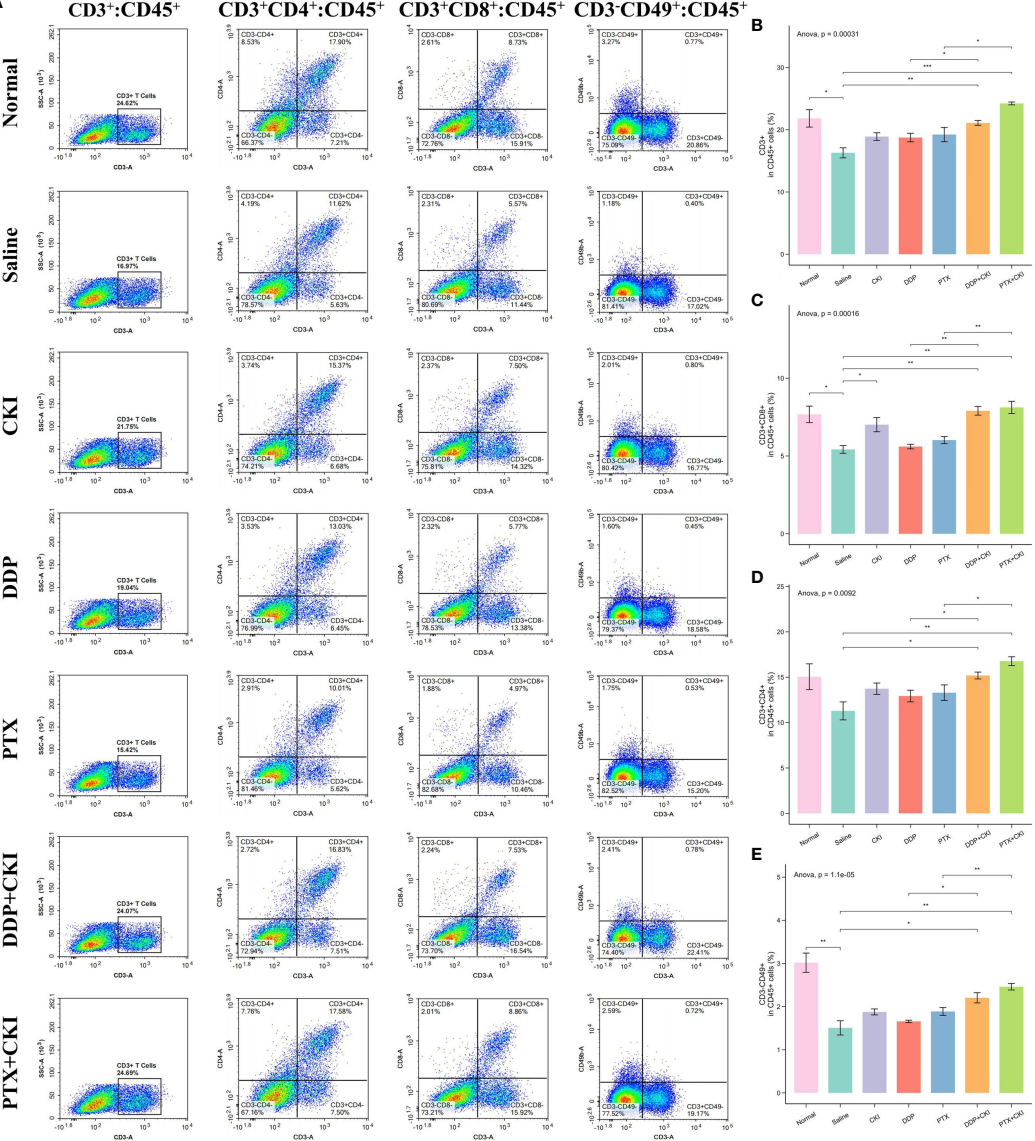
Flow cytometry analysis of immune cell populations within the spleens. **(A)** Representative flow cytometry plots of CD3^+^ T cells, CD4^+^ T cells, CD8^+^ T cells, and NK cells within the spleens of the mice receiving different treatments. The percentage of CD3^+^ T cells **(B)**, CD4^+^ T cells **(C)**, CD8^+^ T cells **(D)**, and NK cells **(E)** within spleens of the mice receiving different treatments. The data are presented as mean ± SEM (n=3). *0.01 < *P* < 0.05, **0.001 < *P* < 0.01, ***P < 0.001.

**Figure 5:**
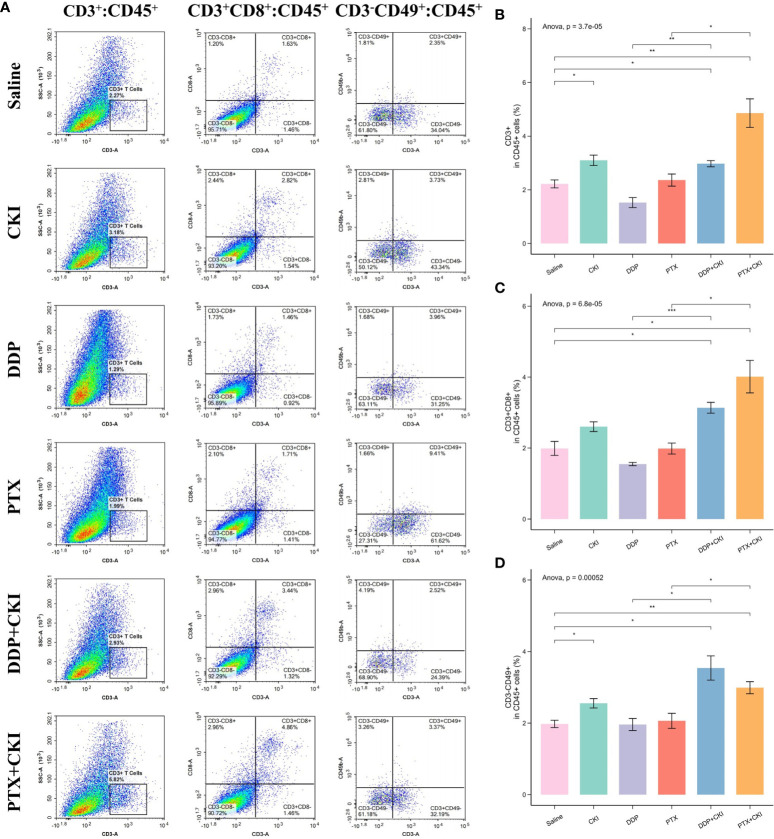
Flow cytometry analysis of immune cell populations within tumors and immune infiltrating analysis based on RNA-seq data. **(A)** Representative flow cytometry plots of CD3^+^ T cells, CD8^+^ T cells, and NK cells within the tumors of the mice receiving different treatments. The percentage of CD3^+^ T cells **(B)**, CD8^+^ T cells **(C)**, and NK cells **(D)** within tumors receiving different treatments. The data are presented as mean ± SEM (n=3). *0.01 < *P* < 0.05, **0.001 < *P* < 0.01, ***P < 0.001.

To investigate the immune responses exerted by CKI *in vivo*, we analyzed the immune cell populations from the spleen and tumor tissues of mice by flow cytometry. For the cell subsets in spleens, the mice treated with saline witnessed a decreased number of immune cells compared to the normal control mice, and the T cell population (CD3^+^), CD8^+^ T lymphocytes (CD3^+^CD8^+^), CD4^+^ T cells (CD3^+^CD4^+^) and NK cells (CD3^-^CD49^+^) of the mice treated with DDP+CKI or PTX+CKI were significantly higher than that of the saline-treated mice (**Figure 4**). Meanwhile, the mice with combination therapy showed a significantly higher number of T lymphocytes and NK cells in spleens compared with the monotherapy-treated mice (**Figure 4**). For the cell subsets in tumors, the mice treated with combination therapy also showed an increased number of immune cells compared to the saline-treated mice, and the CD3^+^ T, CD8^+^ T, and NK cell populations of the mice treated with DDP+CKI or PTX+CKI was significantly higher than that of the saline-treated mice (**Figure 5**). Meanwhile, the mice with combination therapy showed a significantly higher number of tumor-infiltrating CD3^+^ T, CD8^+^ T, and NK cells compared with the monotherapy-treated mice (**Figure 5**).

### Bulk RNA-seq analysis of 4T1 tumor bearing mice

**Figure 6:**
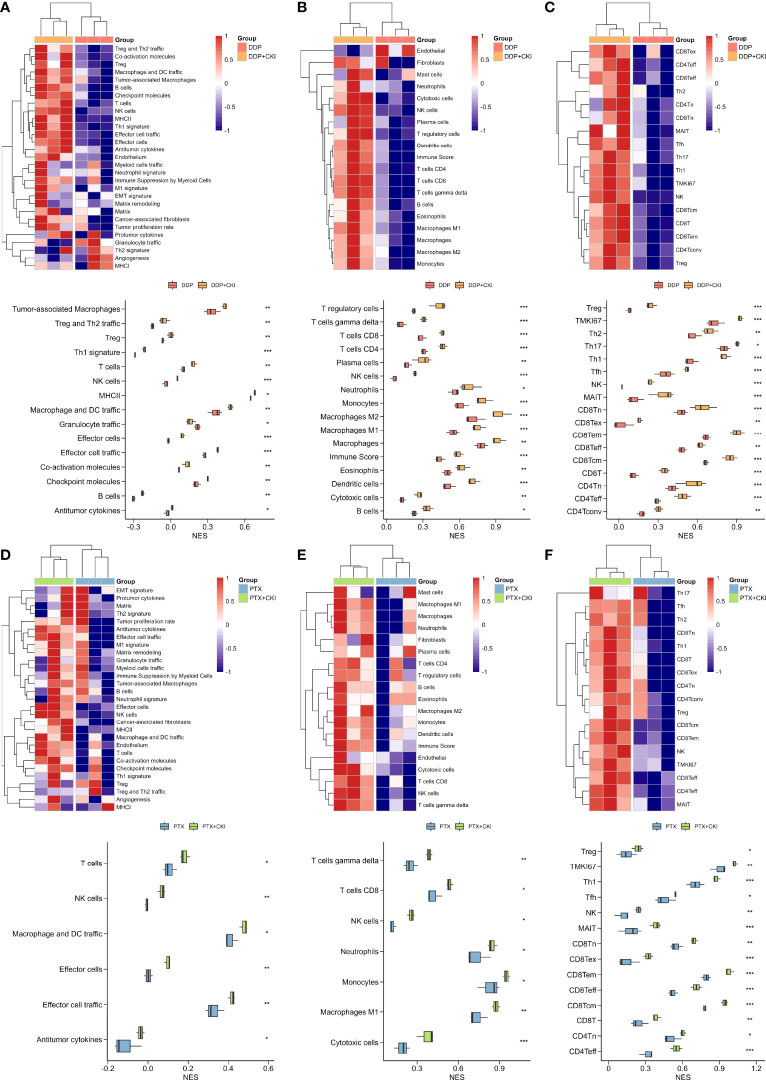
Estimation of immune cell infiltration in tumor tissues based on ssGSEA. Relative abundance of tumor-infiltrating immune cells in the tumor tissues of the 4T1 tumor bearing mice treated with DDP or DDP+CKI, which was calculated based on the signatures developed by Bagaev et al. **(A)**, Jimenez-Sanchez et al. **(B)**, and Sun et al. **(C)**, respectively. Relative abundance of tumor-infiltrating immune cells in the tumor tissues of the 4T1 tumor bearing mice treated with PTX or PTX+CKI, which was calculated based on the signatures developed by Bagaev et al. **(D)**, Jimenez-Sanchez et al. **(E)**, and Sun et al. **(F)**, respectively. The *P* values were generated by the limma package. *0.01 < *P* < 0.05, **0.001 < *P* < 0.01, ***P < 0.001.

The RNA-seq data of the 4T1 mouse treated with DDP, PTX, DDP+CKI and PTX+CKI was shown in **Supplementary Table 5**. We quantified the relative tumor infiltration levels of immune cell types based on transcriptome profiling of tumour tissues by using the ssGSEA approach (implemented in the GSVA package). We used the normalized enrichment score (NES) of every immune cell signature to indicate the relative abundance of each immune cell in the 4T1 tumor-bearing mice. As for the gene signatures developed by Bagaev et al., the mice treated with combination therapy witnessed a remarkablely higher enrichment scores of the signatures associated with cytotoxic T and NK cells, such as effector cells, effector cell traffic, T cells, and NK cells (**Figures 6A, D**). With regard to the gene signatures developed by Jimenez-Sanchez et al., all the mice with DDP+CKI or PTX+CKI treatment showed a remarkablely higher enrichment scores of cytotoxic cells, T cells CD8, and NK cells than the mice with a single chemotherapy (**Figures 6B, E**). Similarly, for the gene signatures developed by Sun et al., the mice in the combination therapy group were also witnessed a significantly higher enrichment scores in CD8^+^ and CD4^+^ T cells than the mice with monotherapy (**Figures 6C, F**). In summary, the results indicated that CKI could promote the cytotoxic immune cell infiltrating into tumor tissues.

### ScRNA-seq analysis of the TME in 4T1 tumor bearing mice

**Figure 7:**
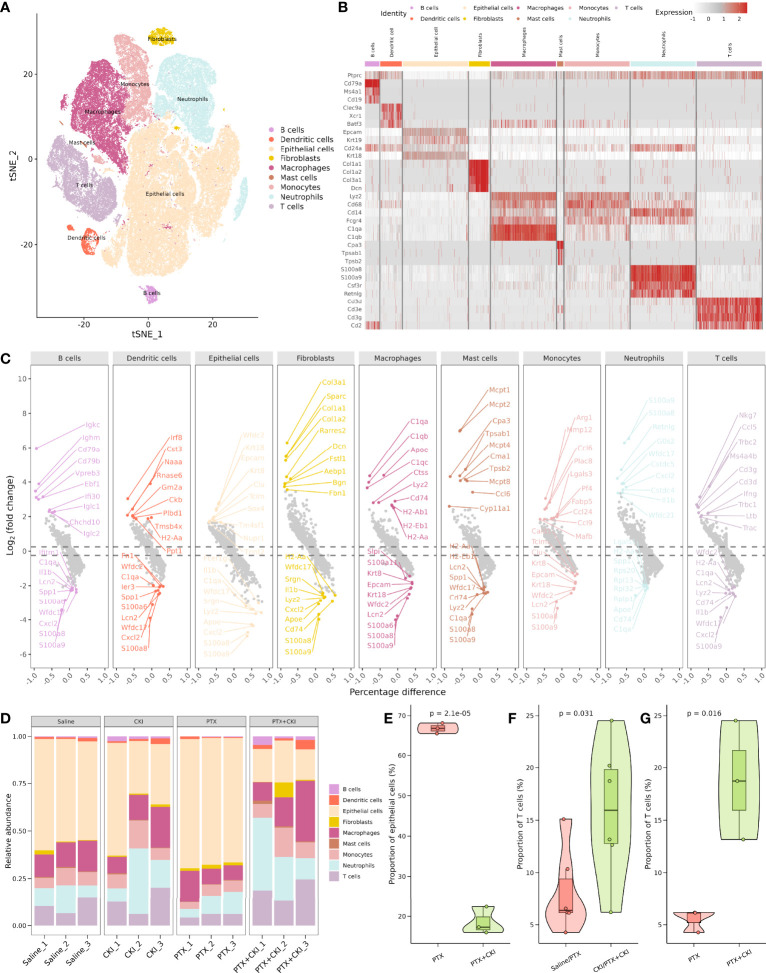
Comprehensive dissection and clustering of single cells from 4T1 mice. **(A)** T-SNE plot within each sample type, color-coded by cell types.**(B)** Heatmap of average expression of canonical marker genes for different cell types. **(C)** Volcano plot showing top 10 up- and down-regulated DEGs for each cell type. **(D)** Difference in the proportion of different cell types. **(D)** Difference in the proportion of different cell types. **(E)** Difference in the proportion of epithelial cells between the PTX+CKI and PTX group. **(F)** Difference in the proportion of T cells between the mice with or without CKI intervention. **(G)** Difference in the proportion of T cells between the PTX+CKI and PTX group.

To characterize the influence of CKI combined with PTX on the TME of TNBC mice, we performed droplet-based scRNA-seq for the mice treated with saline, CKI, PTX and PTX+CKI. In total, we sequenced 174434 cells, with an average of 14536 cells per sample. We detected a total of 32285 genes with an average of 1087 genes and 3263 unique molecular identifiers (UMIs) in each cell (**Supplementary Figure 2A**; **Supplementary Table 2**). After performing quality control based on total UMIs, the number of expressed genes, the percentages of UMIs from mitochondrial genes, ribosomal genes, dissociation-associated genes, and hemoglobin genes, we reserved a total of 17925 genes and 104940 cells, with an average of 1900 genes and 5998 UMIs detected per cell (**Supplementary Figure 2B**; **Supplementary Table 3**). We annotated epithelial cells, neutrophils, macrophages, T cells, monocytes, dendritic cells, fibroblasts, B cells and mast cells through the expression of canonical cell type gene markers. (**Figures 7A–C**; **Supplementary Figures 3**, **4A–C**). Four major cell clusters were epithelial cells (Epcam^+^, Krt18^+^, Krt7^+^), neutrophils (S100a8^+^, S100a9^+^, Csf3r^+^, Retnlg^+^), macrophages (C1qa^+^, C1qb^+^) and T cells (Cd3d^+^, Cd3e^+^, Cd2^+^), including 48634, 15444, 15261 and 12181 cells, respectively. Five minor cell clusters were monocytes (Cd68^+^, Cd14^+^), dendritic cells (Clec9a^+^, Xcr1^+^, Batf3^+^), fibroblasts (Col1a1^+^, Col3a1^+^), B cells (Cd79a^+^, Ms4a1^+^) and mast cells (Cpa3^+^, Tpsab1^+^), including 8541, 1656, 1608, 1112 and 503 cells, respectively. The mice receiving CKI intervention showed a higher percentage of T cells and a lower percentage of epithelial cells compared with the mice without CKI treatment (**Figures 7D–G**, **Supplementary Figure 4D**).

**Figure 8:**
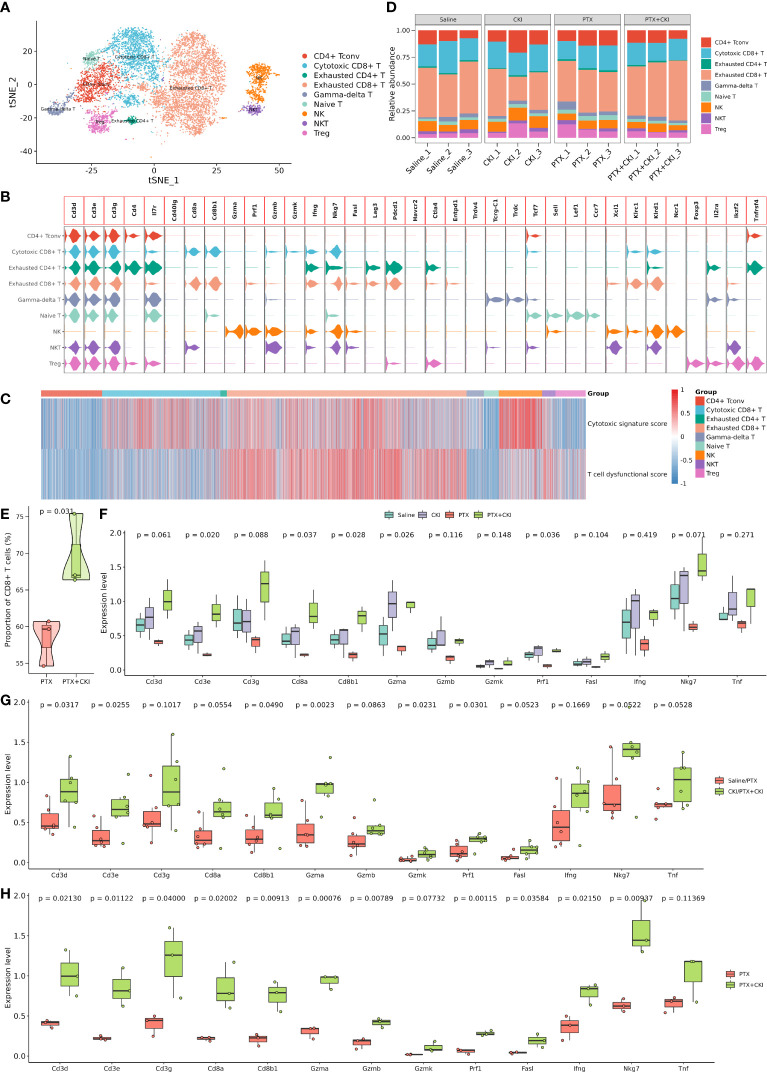
Dissection and clustering of T lymphocytes in 4T1 mice. **(A)** T-SNE plot within each sample type, color-coded by cell types. **(B)** Violin plot of average expression of canonical marker genes for different cell types. **(C)** Heatmap of dysfunctional and cytotoxic effector gene signature scores in T cells. **(D)** Difference in the proportion of different cell types. **(E)** Difference in the proportion of CD8^+^ T cells between the PTX+CKI and PTX group. **(F)** Difference in the expression levels of canonical marker genes for T and cytotoxic T cells. **(G)** Difference in the expression levels of canonical marker genes for T and cytotoxic T cells between the mice with or without CKI intervention. **(H)** Difference in the expression levels of canonical marker genes for T and cytotoxic T cells between the PTX+CKI and PTX group.

The T cell subcluster was further classified into exhausted CD8^+^ T cells, cytotoxic CD8^+^ T cells, conventional CD4^+^ T cells (CD4^+^ Tconv), NK cells, regulatory T cells (Treg), γδ T cells, Naive T cells, NKT cells and exhausted CD4^+^ T cells, including 4788, 2364, 1220, 869, 603, 346, 297, 265 and 128 cells, respectively (**Figures 8A–C**, **Supplementary Figures 5**, **6A–D**). The mice treated with PTX+CKI showed a higher percentage of CD8^+^ T cells compared with the mice treated with PTX, while cytotoxic CD8^+^ T cells had no statistical significance (**Figures 8D, E**, **Supplementary Figure 6E**). Overall, the mice in the PTX+CKI and CKI groups showed a higher average expression of canonical T cell markers (Cd3d, Cd3e, Cd3g), CD8^+^ T cell markers (Cd8a, Cd8b1) and cytotoxic CD8^+^ T cell markers (Gzma, Gzmb, Gzmk, Prf1, Fasl, Ifng, Nkg7, Tnf) than the mice without CKI intervention (**Figures 8F–H**).

**Figure 9:**
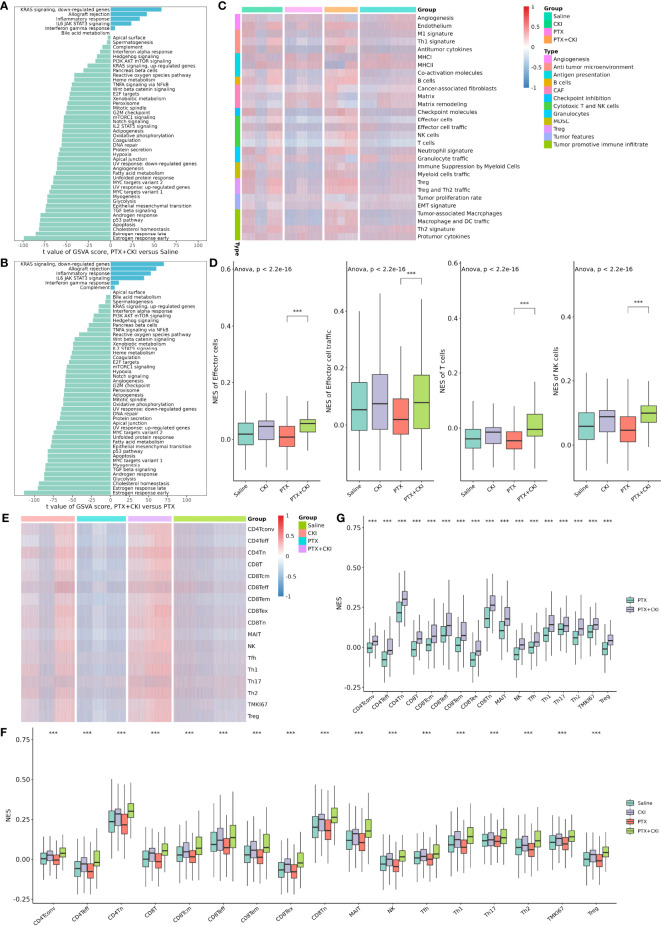
GSVA analysis of single cells from 4T1 mice. **(A)** Differential hallmark pathway activities in the PTX+CKI group versus the saline group. **(B)** Differential hallmark pathway activities in the PTX+CKI group versus the PTX group. **(C)** Heatmap showing enrichment scores (based on the signatures developed by Bagaev et al.) in the four groups. **(D)** Difference in the enrichment scores of the signatures related to cytotoxic T and NK cells among the four groups (based on the signatures developed by Bagaev et al.). **(E)** Heatmap showing T cell enrichment scores (based on the signatures developed by Sun et al.) in the four groups. **(F)** Difference in the T cell enrichment scores (based on the signatures developed by Sun et al.) among the four groups. **(G)** Difference in the T cell enrichment scores (based on the signatures developed by Sun et al.) between the PTX+CKI group and the PTX group. ***P < 0.001.

**Figure 10:**
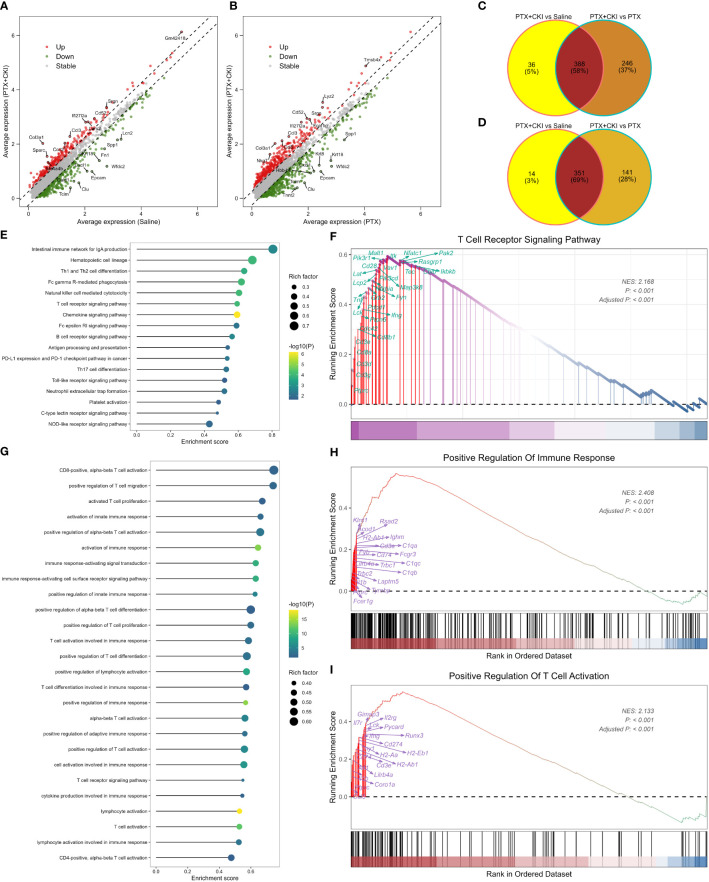
GSEA analysis of single cells from 4T1 mice. **(A)** Volcano plot showing DEGs between the mice treated with PTX+CKI and saline. **(B)** Volcano plot showing DEGs between the mice treated with PTX+CKI and PTX. The red dot represents up-regulated genes (adjusted *P* < 0.01 and log_2_FC > 0.25) and the green dot represents down-regulated genes (adjusted *P* < 0.01 and log_2_FC < -0.25). **(C)** Venn plot showing consistently down-regulated DEGs. **(D)** Venn plot showing consistently up-regulated DEGs. **(E)** Immune-related KEGG pathways enriched in the PTX+CKI group compared with the PTX group. **(F)** T cell receptor signaling pathway enriched in the PTX+CKI group compared with the PTX group. **(G)** Immune-related GO biological processes enriched in the PTX+CKI group compared with the PTX group. **(H)** Positive regulation of immune response enriched in the PTX+CKI group compared with the PTX group. **(I)** Positive regulation of T cell activation enriched in the PTX+CKI group compared with the PTX group.

GSVA analysis based on hallmark gene sets showed that tumor-promoting signaling pathways were inhibited in the PTX+CKI group, such as TGF beta signaling, MYC targets variant 1 and MYC targets variant 2 (PTX+CKI vs. saline and PTX+CKI vs. PTX, **Figures 9A, B**). GSVA analysis based on immune gene sets exhibited that the signatures associated with cytotoxic T and NK cells were activated in the PTX+CKI group versus the PTX group, like effector cells, effector cell traffic, NK cells, T cells (**Figures 9C, D**), CD8Tcm, CD8Teff and CD8Tem (**Figures 9E–G**). GSEA analysis based on KEGG pathways showed that the PTX+CKI group were enriched in T cell-related pathways compared with the PTX group, like T cell receptor signaling pathway, Th1 and Th2 cell differentiation, Th17 cell differentiation, natural killer cell mediated cytotoxicity, and PD-L1 expression and PD-1 checkpoint pathway in cancer (**Figures 10E, F**). GSEA analysis based on GO biological processes showed that the PTX+CKI group were enriched in the biological processes associated with the activation of immune response and T cells, like positive regulation of adaptive and innate immune response, lymphocyte activation involved in immune response, and positive regulation of T cell activation, proliferation, differentiation and migration (PTX+CKI vs. PTX, **Figures 10G–I**). Moreover, we found that the PTX+CKI vs.saline group and the PTX+CKI vs. PTX group shared 58% down-regulated genes and 69% up-regulated genes, implying the potential synergy of CKI with PTX (**Figures 10A–D**).

### CKI inhibits proliferation, clone formation and migration of MDA-MB-231 cells

**Figure 11:**
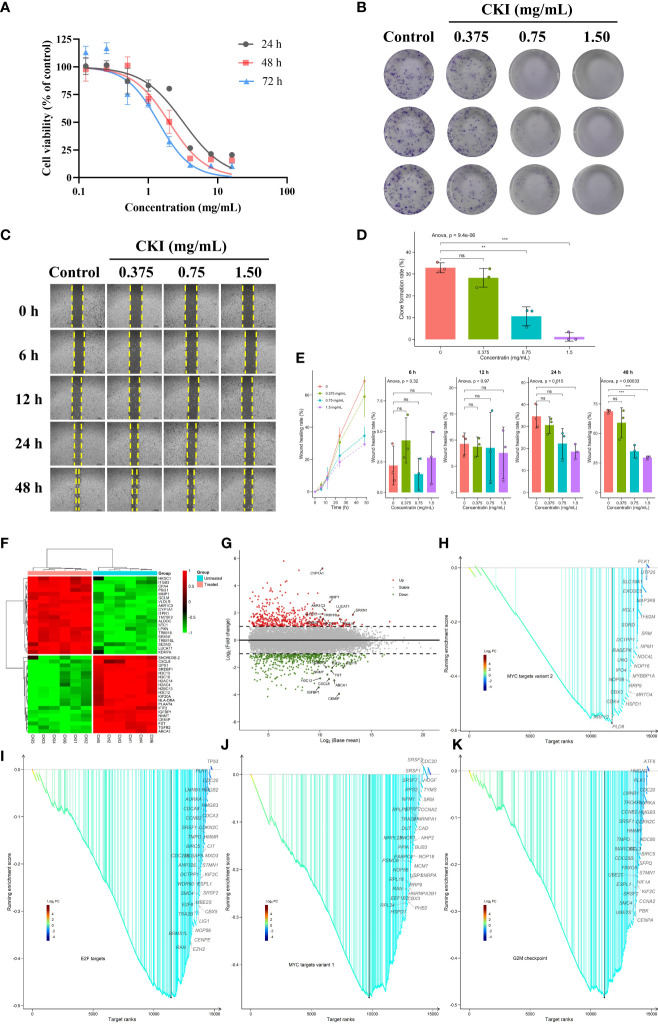
Dose-dependent inhibitory effects of CKI on the proliferation, clone formation and migration of MDA-MB-231 cells. **(A)** The viability of MDA-MB-231 cells treated with different concentrations of CKI for 24, 48, and 72 h, respectively. **(B)** Clone formation images of MDA-MB-231 cells treated with different concentrations of CKI. **(C)** Wound healing images of MDA-MB-231 cells treated with different concentrations of CKI for 6, 12, 24 and 48 h, respectively. **(D)** Bar plot showing clone formation rates. **(E)** Bar plot showing wound healing rates. **(F)** Heatmap of significantly differential genes. Top 20 up- and down-regulated genes in the differential genes with base mean larger than the median value were shown. **(G)** Volcano plot of significantly differential genes. The red dot represents up-regulated genes (adjusted *P* < 0.05 and log_2_FC > 1) and the green dot represents down-regulated genes (adjusted *P* < 0.05 and log_2_FC < -1). Top 10 up- and down-regulated differential genes were labeled. **(H)** GSEA analysis of genes involved in MYC targets variant 2 pathway. **(I)** GSEA analysis of genes involved in E2F targets pathway. **(J)** GSEA analysis of genes involved in MYC targets variant 1 pathway. **(K)** GSEA analysis of genes involved in G2M checkpoint pathway. Data represents mean ± SD (n=3 per group). *0.01 < *P* < 0.05, **0.001 < *P* < 0.01, ***P < 0.001. ns, non-significant.

**Figure 12:**
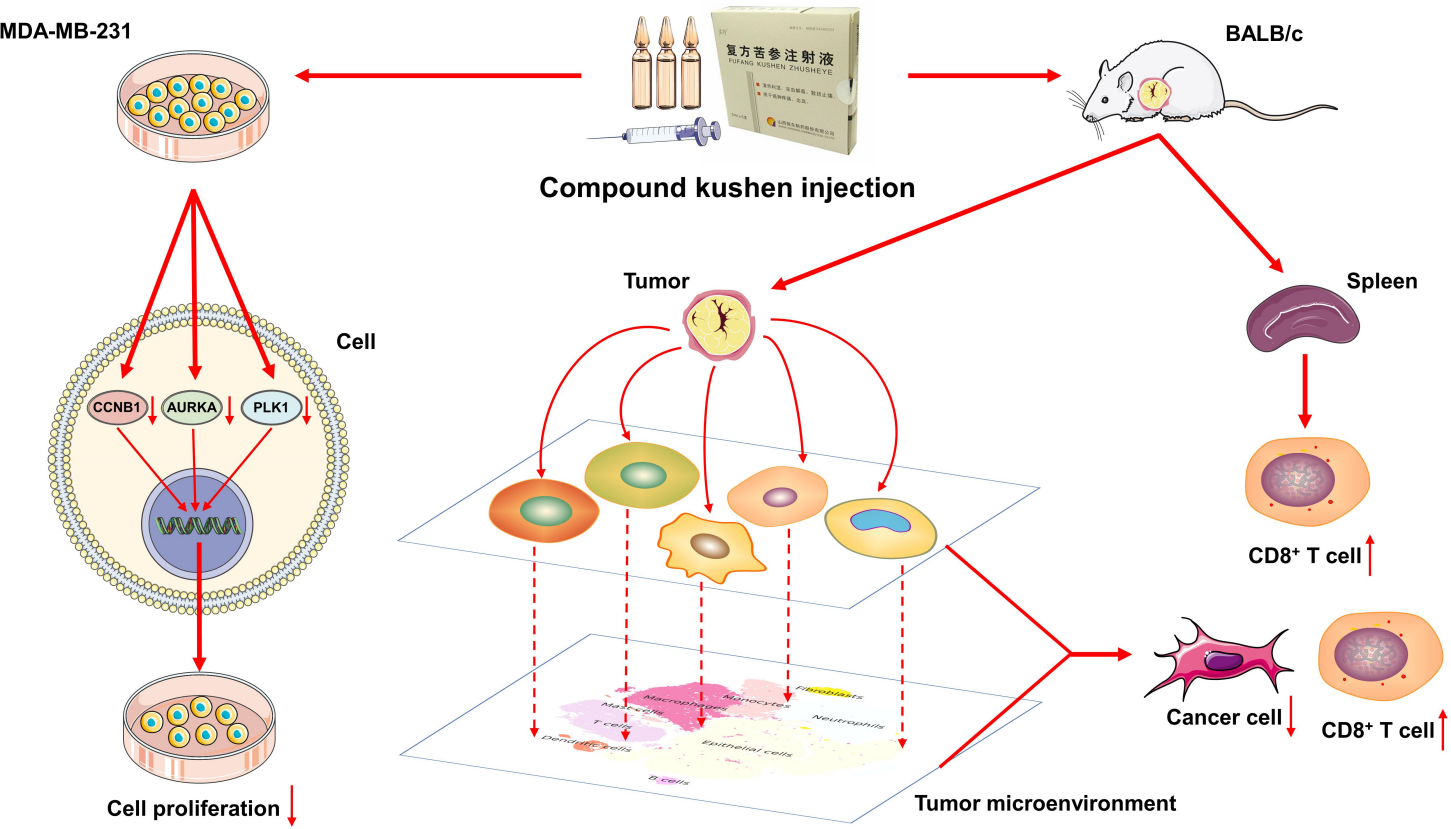
Schematic diagram shows the effects of CKI on TNBC *in vitro* and *in vivo*.

We assessed the effect of CKI on the viability of human breast cancer MDA-MB-231 cells, and the results presented that CKI inhibited the growth of MDA-MB-231 cells in a dose-dependent manner. After 24, 48, and 72 h of CKI treatment, the IC_50_ values of CKI on MDA-MB-231 cells were 2.62 ± 0.52 mg/mL, 1.89 ± 0.10 mg/mL, and 1.419 ± 0.39 mg/mL, respectively (**Figure 11A**; **Supplementary Figure 4**). Based on the IC_50_ values and cellular state, the MDA-MB-231 cells with 2 mg/mL CKI treatment for 48 h were chosen for the RNA sequencing. The clone formation assay also indicated that CKI strongly repressed the proliferation of MDA-MB-231 cells (**Figures 11B, D**). Wound-healing assay showed that the wound closure rate of the control group was higher than that of the CKI-treated group, and 1.5 mg/ml CKI obviously inhibited the migration of MDA-MB-231 cells at 24 and 28 h (**Figures 11C, E**). The RNA-seq data of CKI-perturbed MDA-MB-231 cells was shown in **Supplementary Table 6**. The results of RNA-seq data analysis for the CKI-perturbed cell line samples and control samples were shown in **Figures 11F–K**. We found a total of 1024 significantly differential genes (540 down-regulated and 484 up-regulated), which contained 853 protein coding genes and 134 long non-coding RNAs (lncRNAs) (**Figures 11F, G**; **Supplementary Table 4**). Analysis of hallmark pathway gene signatures highlighted that proliferation-related pathways like MYC targets variant 2 (**Figure 11H**), E2F targets (**Figure 11I**), MYC targets variant 1 (**Figure 11J**), and G2M checkpoint (**Figure 11K**) were significantly down-regulated in the CKI treatment group compared with the control group. Collectively, these results highlighted the antitumor functions of CKI *in vitro*. As a whole, the schematic diagram showing the overall effects of CKI on TNBC *in vitro* and *in vivo* (**Figure 12**).

## Discussion

Chemotherapy is a standard therapeutic regimen to treat triple-negative breast cancer (TNBC); nevertheless, chemotherapy alone does not bring more improvement of clinical benefits and often leads to drug resistance in patients. Cancer immunotherapies that target tumor-specific T cells, especially reactivating CD8^+^ T cells to promote anti-tumor immunity, have successfully provided a clinical benefit to cancer patients (22, 57–59). Recently, CD8 status has been proposed to determine which TNBC patients have a higher possibility to benefit from immunotherapy (9). However, the strategies attempting to reactivate CD8^+^ T cells tend to have possible drug toxicities and high costs, and targeting CD8^+^ T cells alone does not provide enough efficacy for successful cancer treatment considering the complexity of the immunosuppressive microenvironment (9, 60). Moreover, the efficacy of immunotherapy varies dramatically across cancer types, and only a minor subset of TNBC patients is associated with immunotherapy benefits (8, 10, 22). Thus, it becomes more important to develop newer approaches for breast cancer treatment using the existing chemotherapy drugs.

CKI has been clinically approved by NMPA for treating cancer-induced pain (25), and CKI alone or it combined with chemotherapy or radiotherapy has been widely used and improved therapeutic and prognostic benefits (29, 31). Meanwhile, previous research has reported that CKI could remodel the tumor immune microenvironment of hepatocellular carcinoma (HCC) *via* modulating the function of macrophages and CD8^+^ T cells (24). Given the immunoregulatory roles of CKI on HCC and its broad-spectrum anticancer activity, we attempted to explore whether the combination of CKI and chemotherapy could enhance the anti-tumor immunity in TNBC. Numerous studies have found that CKI exerts significant inhibition on the migration and invasion of MDA-MB-231 cells, and it also induces both apoptosis and cell cycle arrest (56, 61–63). In addition, the energy metabolism and DNA repair pathways of MDA-MB-231 cells are reduced after CKI intervention (62). Our previous research also reveals potential immunotherapy biomarkers of CKI on TNBC by applying transcriptome data mining (38). Collectively, existing clinical and experimental results indicate that CKI holds a great promise as an adjuvant for anticancer immunotherapy.

In this study, we assessed the antitumor efficacy and toxicity of DDP+CKI or PTX+CKI in a 4T1 murine breast cancer model using BALB/c mice. The results showed that the combination of CKI with chemotherapy synergistically suppressed tumor growth without obvious organ toxicity. The flow cytometry analysis showed an elevated percentage of CD3^+^, CD4^+^, CD8^+^ T lymphocytes and NK cells in the spleens and tumors of combination therapy-treated mice, suggesting the immunomodulatory effects of CKI when it was combined with chemotherapy. Based on the bulk RNA-seq data of the tumor tissues of 4T1 mice, we adopted different curated gene lists for immune cell infiltrating analysis to maximally ensure the reliability of our research. The results demonstrated that the gene sets correlated with T, cytotoxic CD8^+^ T and NK cells were significantly activated in the combination therapy group versus the monotherapy group. Notably, all the results support that the cytotoxic T and NK cells in the mice treated with combination therapy were significantly activated compared to the mice treated with a single chemotherapy.

The scRNA-seq analysis for the TME of 4T1 tumor bearing mice showed that the mice in the PTX+CKI group had a higher percentage of total T lymphocytes and CD8^+^ T cells than the PTX-treated mice. Meanwhile, the mice in the PTX+CKI and CKI groups showed a higher average expression of canonical marker genes of T, CD8^+^ T and cytotoxic CD8^+^ T cells than the mice without CKI intervention. However, although the number of cytotoxic CD8^+^ T cells in the PTX+CKI group was much higher than that in the PTX group, the percentage of cytotoxic CD8^+^ T cells had no statistical significance between the two groups. Two possible reasons may contribute to such results: on the one hand, the combination of CKI and PTX may only increase the number of cytotoxic CD8^+^ T cells in TME without changing the ratio of cytotoxic CD8^+^ T cells to total T lymphocytes; on the other hand, the small sample size limited statistical power, so more scRNA-seq samples in a large number of animals are needed to further observe the results. Furthermore, we found that the pathways and biological processes associated with the activation of immune response, lymphocytes, T cell, cytotoxic T cells and NK cells were enriched in the PTX+CKI group versus the PTX group. Altogether, CKI could promote the cytotoxic immune cell infiltrating into tumor tissues, eventually improving the therapeutic outcomes of DDP and PTX. Analysis of bulk RNA-seq data on CKI-treated MDA-MB-231 cells displayed that proliferation-related pathways like MYC targets, G2M checkpoint and E2F targets were significantly down-regulated in the CKI-treated tumor cells compared with the control cells. All these results highlighted the antitumor functions of CKI *in vitro* and *in vivo*. In summary, this study found that CKI modulated TME while DDP or PTX directly targeted tumor cells, and these distinct modes of action promoted anticancer activity and achieved significant tumor suppression.

To our knowledge, this is the first study that employs the scRNA-seq technology to investigate the antitumor effects of Chinese patent medicines. Our findings may be useful in further developing a combination of drugs with high efficiency and low toxicity to control cancer growth in TNBC, and our analytic methods would lay the foundation for further dissecting the mechanisms of compound medicines on TME. However, our study was limited to the small sample size, which may reduce the statistical significance of our results. Further studies in a large number of samples are needed to deeply investigate the immunoregulatory mechanisms of CKI in combination with chemotherapy.

## Conclusions

In conclusion, the combination of CKI and chemotherapy might provide a higher efficiency and lower toxicity strategy than a single chemotherapy drug for TNBC. Our study provides evidence that CKI combined with chemotherapy triggers effective antitumor immunity by activating immune cells in a murine breast cancer model.

## Data availability statement

The raw RNA-seq data of MDA-MB-231 cells reported in this paper have been deposited in the Genome Sequence Archive [1] in National Genomics Data Center [2], China National Center for Bioinformation / Beijing Institute of Genomics, Chinese Academy of Sciences (GSA-Human: HRA003211) that are publicly accessible at https://ngdc.cncb.ac.cn/gsa-human.The raw RNA-seq and scRNA-seq data of 4T1 tumor bearing mice reported in this paper have been deposited in the Genome Sequence Archive [1] in National Genomics Data Center [2], China National Center for Bioinformation / Beijing Institute of Genomics, Chinese Academy of Sciences (GSA: CRA008473; CRA008535) that are publicly accessible at https://ngdc.cncb.ac.cn/gsa.[1] The Genome Sequence Archive Family: Toward Explosive Data Growth and Diverse Data Types. Genomics, Proteomics & Bioinformatics 2021, 19(4):578-583. https://doi.org/10.1016/j.gpb.2021.08.001 [PMID=34400360][2] Database Resources of the National Genomics Data Center, China National Center for Bioinformation in 2022. Nucleic Acids Res 2022, 50(D1):D27-D38. https://doi.org/10.1093/nar/gkab951 [PMID=34718731].

## Ethics statement

All animal experimental procedures were performed in accordance with the NIH Guide for the Care and Use of Laboratory Animals and were approved by the Animal Care and Protection Committee of Beijing University of Chinese Medicine (No: BUCM- 4-2021032001-1088).

## Author contributions

XL and JW conceived, designed, and performed the research. XL analyzed the data and wrote and revised the manuscript. JW, HL, ZZ, and YW supervised the overall study. XL, MB, HYL, PY, CW, and ZH performed the experiments. FG provided computational resources for omics data analysis. XD, FG, ZHZ, SL, JZ, RY, WQ, WW, AH, and LS provided experimental assistance for molecular and animal work. All authors contributed to the article and approved the submitted version.

## Funding

The study was financially supported by National Natural Science Foundation of China (Grant nos. 82074284) and Innovative Team Project for Evaluation of Safety of Mongolian Medicine (Grant nos. MY20190003).

## Conflict of interest

RY, WQ, and WW are employed by the company Beijing Zhendong Pharmaceutical Research Institute Co., Ltd. XD and ZhZ are employed by the company Beijing Zest Bridge Medical Technology Inc. These authors’ involvement with the study was described in author contributions.

The remaining authors declare that the research was conducted in the absence of any commercial or financial relationships that could be construed as a potential conflict of interest.

The reviewer LZ declared a shared affiliation with the author PY to the handling editor at time of review.

## Publisher’s note

All claims expressed in this article are solely those of the authors and do not necessarily represent those of their affiliated organizations, or those of the publisher, the editors and the reviewers. Any product that may be evaluated in this article, or claim that may be made by its manufacturer, is not guaranteed or endorsed by the publisher.

## Supplementary material

Supplementary Figure 1 UHPLC-Q-Exactive-MS analysis of CKI. **(A)** Chromatographic fingerprints of CKI in the positive ion mode. **(B)** Chromatographic fingerprints of CKI in the negative ion mode.

Supplementary Figure 2 Quality control of the samples detected by scRNA-seq. **(A)** Distribution for gene, UMI, mitochondrial gene percentage, ribosomal gene percentage and hemoglobin gene percentage before filtering. **(B)** Distribution for gene, UMI, mitochondrial gene percentage, ribosomal gene percentage and hemoglobin gene percentage after filtering.

Supplementary Figure 3 T-SNE plot of representative marker genes for major cell types.

Supplementary Figure 4 Additional figures for dissection and clustering of major cell types. **(A)** UMAP plot within each sample type, color-coded by cell types. **(B)** Violin plot of average expression of canonical marker genes for different cell types. **(C)** Dot plot of average expression of canonical marker genes for different cell types. **(D)** Average proportion of cells derived from each sample, color-coded by samples.

Supplementary Figure 5 T-SNE plot of representative marker genes for T cell populations.

Supplementary Figure 6 Additional figures for dissection and clustering of T cell populations. **(A)** UMAP plot within each sample type, color-coded by cell types. **(B)** Volcano plot showing top 10 up- and down-regulated DEGs for each cell type. **(C)** Heatmap of average expression of canonical marker genes for different cell types. **(D)** Dot plot of average expression of canonical marker genes for different cell types. **(E)** Average proportion of cells derived from each sample, color-coded by samples.

Supplementary Figure 7 Additional figures for dose-dependent inhibitory effects of CKI on the proliferation of MDA-MB-231 cells. **(A)** Line chart showing the correlation between cell viability and treatment duration. **(B)** Difference in the viability of MDA-MB-231 cells treated with different concentrations of CKI for 24, 48, and 72 h, respectively. Data represents mean ± SD (n=3 per group). *0.01 < *P* < 0.05, **0.001 < *P* < 0.01, ****P* < 0.001, ns, non-significant.
