# Supplementary figures and images for: Single-cell RNA-sequencing uncovers compound kushen injection synergistically improves the efficacy of chemotherapy by modulating the tumor environment of breast cancer
Source: Front Immunol. 2022 Oct 31;13:965342. doi: 10.3389/fimmu.2022.965342 (PMC9660330; doi:10.3389/fimmu.2022.965342)

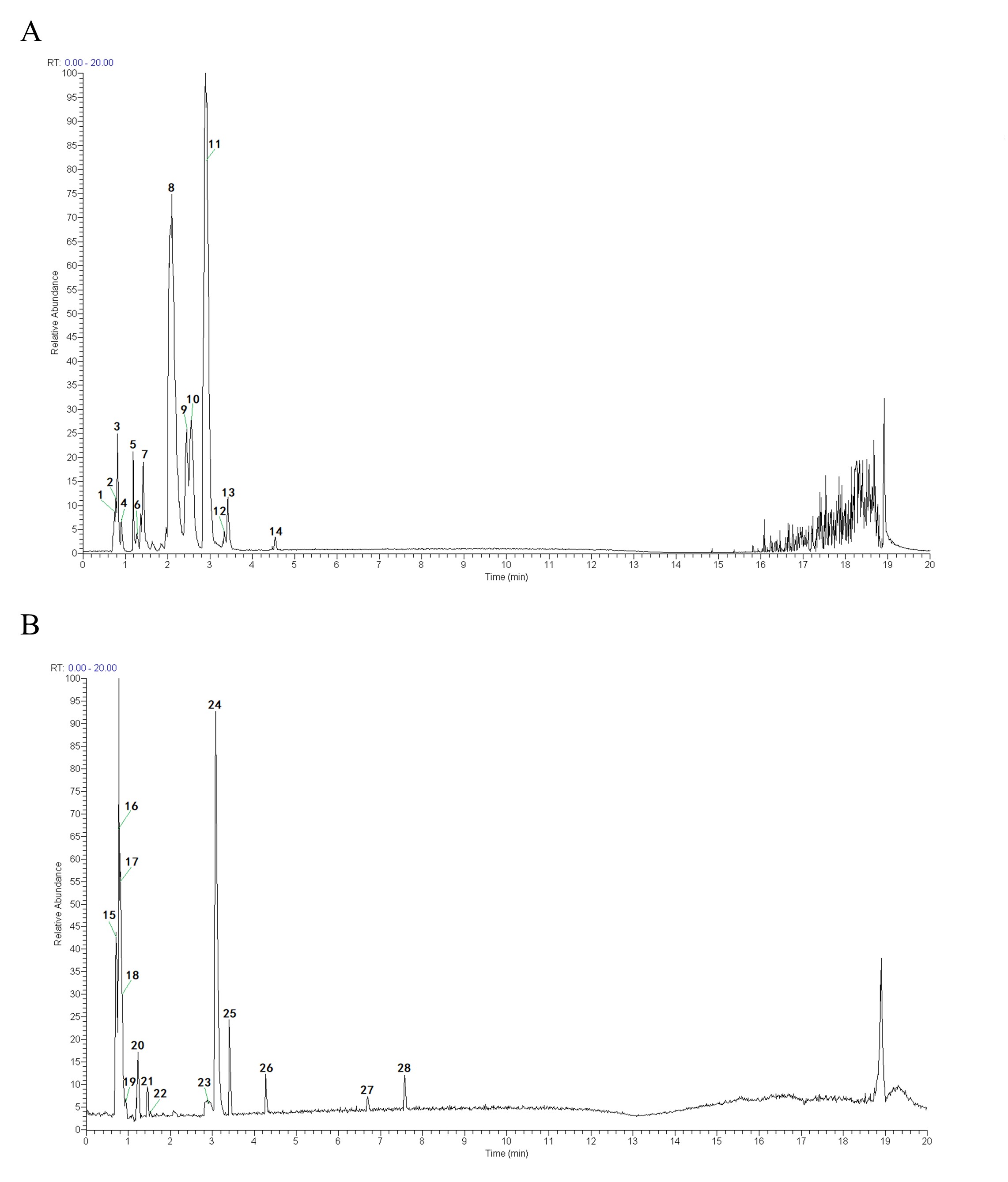

Supplement: Supplementary Figure 1 — UHPLC-Q-Exactive-MS analysis of CKI. (A) Chromatographic fingerprints of CKI in the positive ion mode. (B) Chromatographic fingerprints of CKI in the negative ion mode. [file Image_1.jpeg]

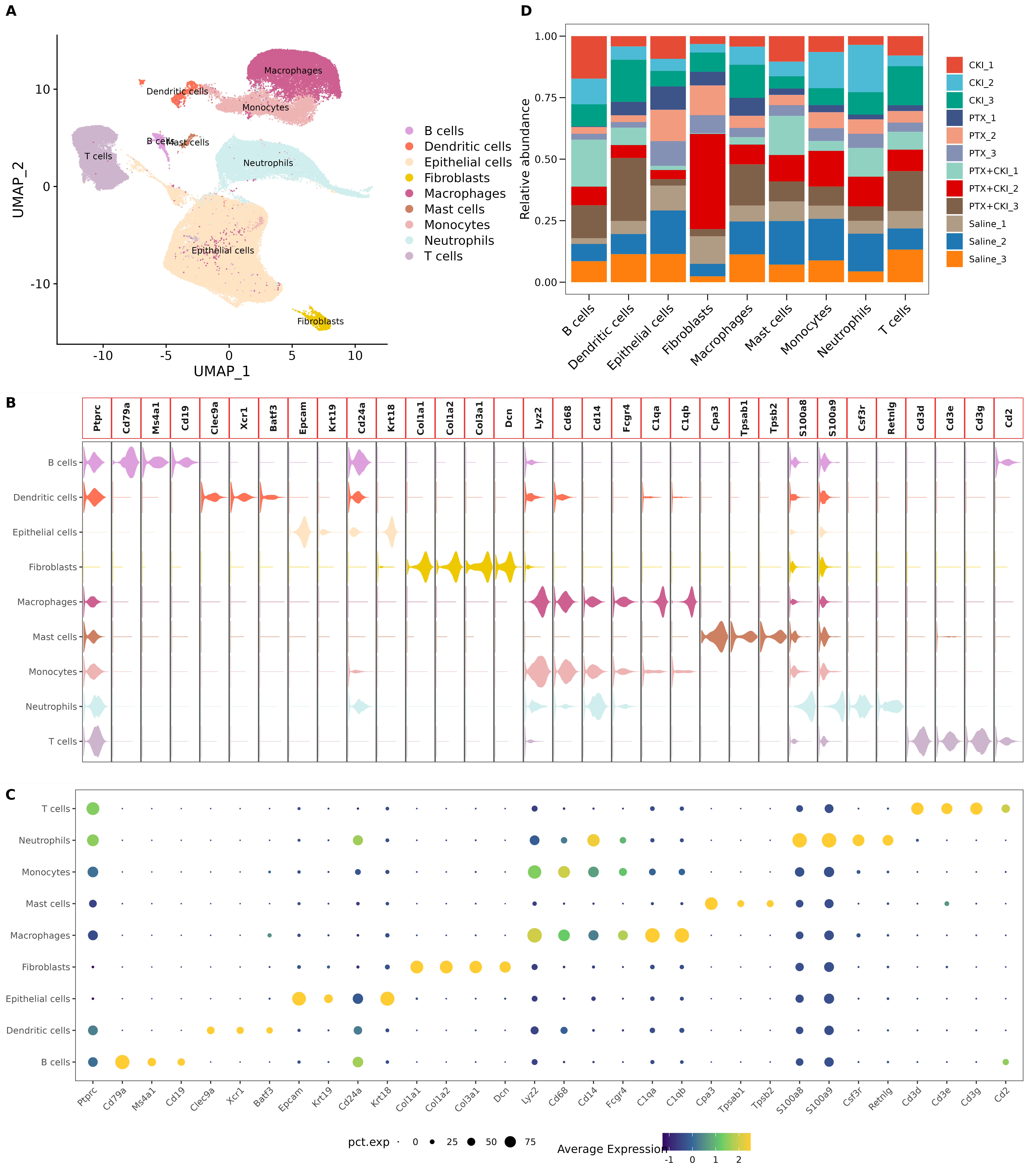

Supplement: Supplementary Figure 4 — Additional figures for dissection and clustering of major cell types. (A) UMAP plot within each sample type, color-coded by cell types. (B) Violin plot of average expression of canonical marker genes for different cell types. (C) Dot plot of average expression of canonical marker genes for different cell types. (D) Average proportion of cells derived from each sample, color-coded by samples. [file Image_4.jpeg]

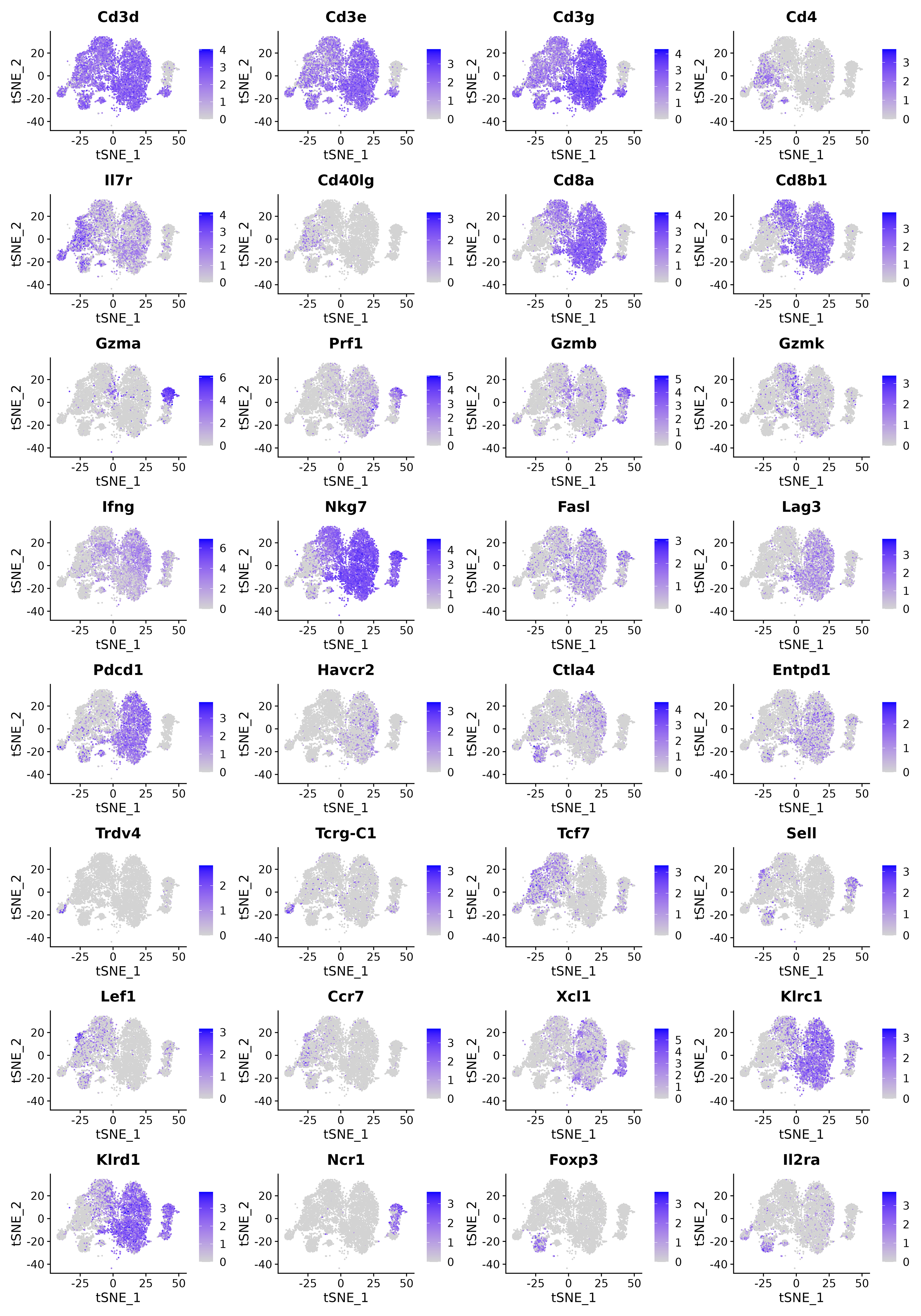

Supplement: Supplementary Figure 5 — T-SNE plot of representative marker genes for T cell populations. [file Image_5.jpeg]

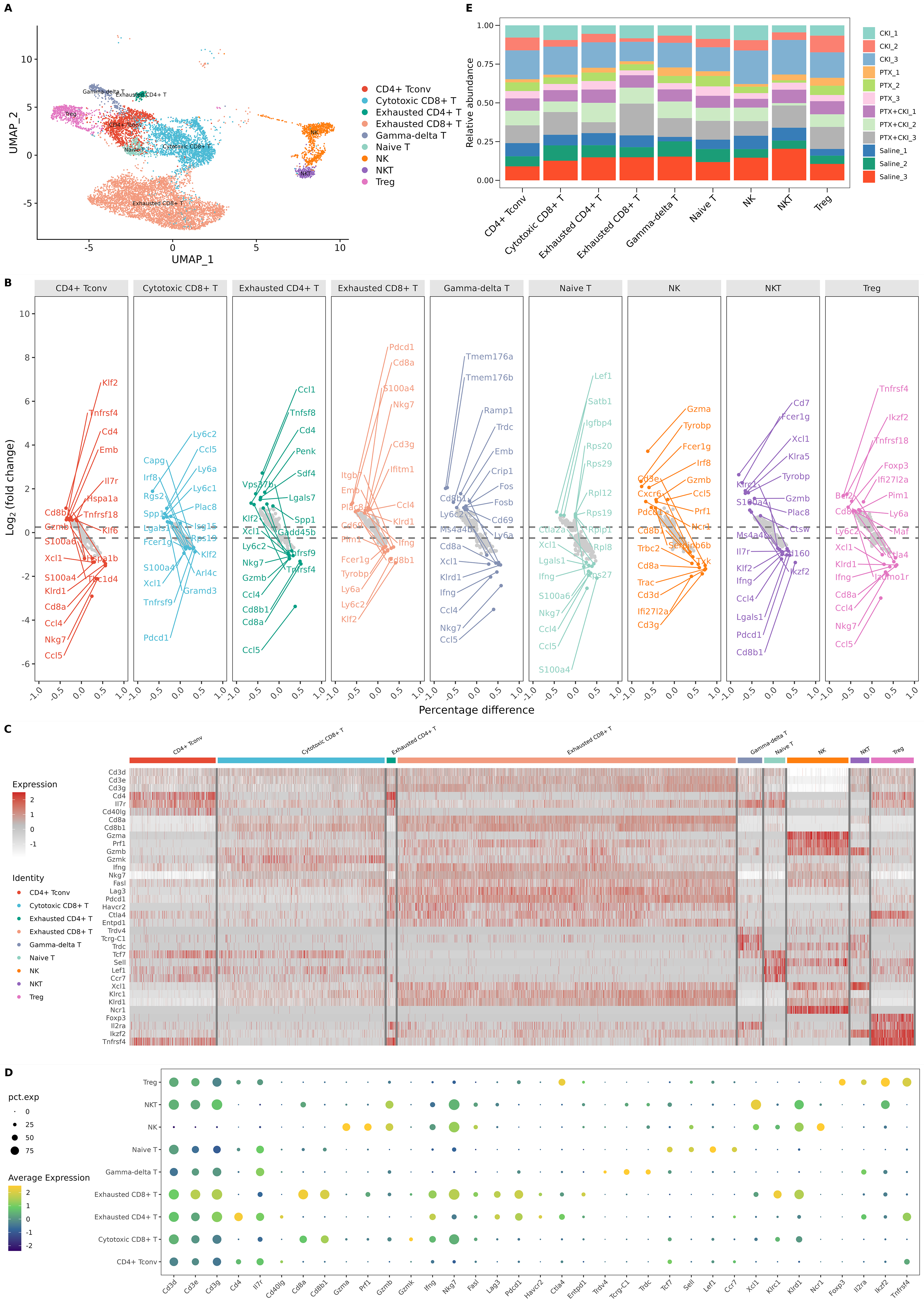

Supplement: Supplementary Figure 6 — Additional figures for dissection and clustering of T cell populations. (A) UMAP plot within each sample type, color-coded by cell types. (B) Volcano plot showing top 10 up- and down-regulated DEGs for each cell type. (C) Heatmap of average expression of canonical marker genes for different cell types. (D) Dot plot of average expression of canonical marker genes for different cell types. (E) Average proportion of cells derived from each sample, color-coded by samples. [file Image_6.jpeg]

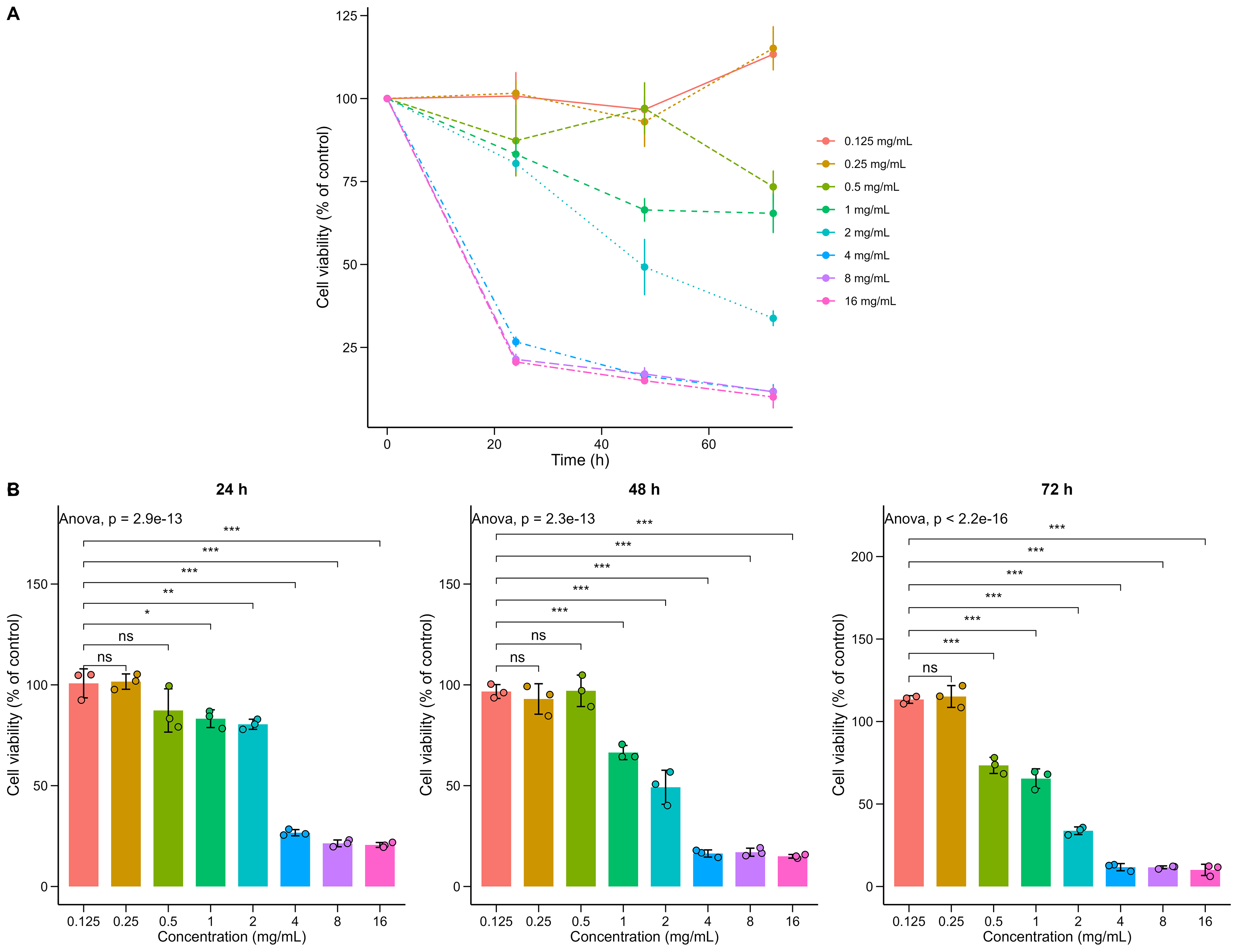

Supplement: Supplementary Figure 7 — Additional figures for dose-dependent inhibitory effects of CKI on the proliferation of MDA-MB-231 cells. (A) Line chart showing the correlation between cell viability and treatment duration. (B) Difference in the viability of MDA-MB-231 cells treated with different concentrations of CKI for 24, 48, and 72 h, respectively. Data represents mean ± SD (n=3 per group). *0.01 < P < 0.05, **0.001 < P < 0.01, ***P < 0.001, ns, non-significant. [file Image_7.png]
