# Supplementary material for: Single-cell RNA-sequencing uncovers compound kushen injection synergistically improves the efficacy of chemotherapy by modulating the tumor environment of breast cancer
Source: Front Immunol. 2022 Oct 31;13:965342. doi: 10.3389/fimmu.2022.965342 (PMC9660330; doi:10.3389/fimmu.2022.965342)
Supplement: Supplementary file 8 [file Table_1.docx]

**Supplementary Table 1. Compounds identified from CKI by UHPLC-Q-Exactive-MS.**

| **No.** | **Compound** | **RT (min)** | **m/z** | **Adduct** | **Formula** | **PubChem CID** | **Classification** |
| --- | --- | --- | --- | --- | --- | --- | --- |
| 1 | Choline | 0.75 | 104.1076 | [Cat]^+^ | C_5_H_14_NO^+^ | 305 | Organic nitrogen compounds |
| 2 | Mannitol | 0.79 | 183.0864 | [M+H]^+^ | C_6_H_14_O_6_ | 6251 | Organic oxygen compounds |
| 3 | 2-Amino-5-Carbamimidoylsulfanylpentanoic Acid | 0.82 | 116.0708 | [M+H-CH_4_N_2_S]^+^ | C_6_H_13_N_3_O_2_S | 53394341 | Organic acids and derivatives |
| 4* | N-Methylcytisine | 0.91 | 205.1336 | [M+H]^+^ | C_12_H_16_N_2_O | 234566 | Alkaloids and derivatives |
| 4* | Macrozamin | 0.93 | 407.1271 | [M+Na]^+^ | C_13_H_24_N_2_O_11_ | 9576780 | Organic oxygen compounds |
| 5* | N-Methylcytisine | 1.21 | 205.1331 | [M+H]^+^ | C_12_H_16_N_2_O | 234566 | Alkaloids and derivatives |
| 6* | Macrozamin | 1.26 | 407.1271 | [M+Na]^+^ | C_13_H_24_N_2_O_11_ | 9576780 | Organic oxygen compounds |
| 7 | Tetracaine | 1.44 | 265.1907 | [M+H]^+^ | C_15_H_24_N_2_O_2_ | 5411 | Benzenoids |
| 8* | Matrine | 2.13 | 249.1963 | [M+H]^+^ | C_15_H_24_N_2_O | 91466 | Alkaloids and derivatives |
| 9* | Sophoridine | 2.49 | 249.1956 | [M+H]^+^ | C_15_H_24_N_2_O | 165549 | Alkaloids and derivatives |
| 10* | Sophocarpine | 2.58 | 247.1803 | [M+H]^+^ | C_15_H_22_N_2_O | 115269 | Alkaloids and derivatives |
| 11* | Oxysophocarpine | 2.87 | 263.1757 | [M+H]^+^ | C_15_H_22_N_2_O_2_ | 161544 | Alkaloids and derivatives |
| 11* | Sophoranol | 2.90 | 265.1909 | [M+H]^+^ | C_15_H_24_N_2_O_2_ | 12442899 | Alkaloids and derivatives |
| 11* | Oxymatrine | 2.91 | 265.1907 | [M+H]^+^ | C_15_H_24_N_2_O_2_ | 114850 | Alkaloids and derivatives |
| 12 | Milnacipran | 3.34 | 247.1804 | [M+H]^+^ | C_15_H_22_N_2_O | 65833 | Benzenoids |
| 13 | Hydroxylupanine | 3.43 | 265.1901 | [M+H]^+^ | C_15_H_24_N_2_O_2_ | 73404 | Alkaloids and derivatives |
| 14 | 3-(2,6-Dimethylmorpholin-4-Yl)-N-Phenylpropanamide | 4.54 | 263.1755 | [M+H]^+^ | C_15_H_22_N_2_O_2_ | 2901294 | Organic acids and derivatives |
| 15 | NA | 0.72 | 112.9845 | NA | NA | NA | NA |
| 16 | Hexitol | 0.78 | 181.0703 | [M-H]^-^ | C_6_H_14_O_6_ | 453 | Organic oxygen compounds |
| 17 | (3R,4R,5R)-1,3,4,5,6-pentahydroxyhexan-2-one | 0.80 | 215.0315 | [M+Cl]^-^ | C_6_H_12_O_6_ | 90008 | Organic oxygen compounds |
| 18 | (2S,3S,4R)-2,3,4,5-tetrahydroxypentanal | 0.86 | 149.0440 | [M-H]^-^ | C_5_H_10_O_5_ | 65550 | Organic oxygen compounds |
| 19 | Malioxamycin | 1.02 | 133.0126 | [M-H-C_5_H_10_N_2_O]^-^ | C_9_H_16_N_2_O_6_ | 175396 | Organic acids and derivatives |
| 20 | L-Pyroglutamic acid | 1.25 | 128.0338 | [M-H]^-^ | C_5_H_7_NO_3_ | 7405 | Organic acids and derivatives |
| 21 | Methylmalonic acid | 1.48 | 117.0176 | [M-H]^-^ | C_4_H_6_O_4_ | 487 | Organic acids and derivatives |
| 22 | Meglutol | 1.55 | 161.0443 | [M-H]^-^ | C_6_H_10_O_5_ | 1662 | Lipids and lipid-like molecules |
| 23 | NA | 2.90 | NA | NA | NA | NA | NA |
| 24 | Piscidic acid | 3.06 | 255.0498 | [M-H]^-^ | C_11_H_12_O_7_ | 120693 | Phenylpropanoids and polyketides |
| 25 | N-benzylnaphthalene-2-sulfonamide | 3.38 | 232.1178 | [M-H-SO_2_]^-^ | C_17_H_15_NO_2_S | 873831 | Benzenoids |
| 26 | 2,3-Dihydrobenzofuran-5-acetic acid | 4.25 | 177.0543 | [M-H]^-^ | C_10_H_10_O_3_ | 2737455 | Organoheterocyclic compounds |
| 27 | NA | 6.70 | NA | NA | NA | NA | NA |
| 28* | Trifolirhizin | 7.58 | 491.1176 | [M+HCO_2_]^-^ | C_22_H_22_O_10_ | 442827 | Phenylpropanoids and polyketides |

*The compounds unambiguously identified with authentic standards comparison.
