# Supplementary material for: Single-cell RNA-sequencing uncovers compound kushen injection synergistically improves the efficacy of chemotherapy by modulating the tumor environment of breast cancer
Source: Front Immunol. 2022 Oct 31;13:965342. doi: 10.3389/fimmu.2022.965342 (PMC9660330; doi:10.3389/fimmu.2022.965342)
Supplement: Supplementary file 9 [file Table_2.docx]

**Supplementary Table 2. Metric information for the samples subjected to scRNA-seq (generated by the Cellranger software).**

| **Sample ID** | **Group** | **scRNA-seq platform** | **Chemistry version** | **Estimated number of cells** | **Median UMI counts per cell** | **Median genes per cell** |
| --- | --- | --- | --- | --- | --- | --- |
| Saline_1 | Saline | 10x Genomics Chromium | Single Cell 3' v3 | 12,693 | 5,871 | 1,788 |
| Saline_2 | Saline | 10x Genomics Chromium | Single Cell 3' v3 | 24,949 | 2,962 | 1,128 |
| Saline_3 | Saline | 10x Genomics Chromium | Single Cell 3' v3 | 15,494 | 4,724 | 1,592 |
| CKI_1 | CKI | 10x Genomics Chromium | Single Cell 3' v3 | 12,323 | 4,415 | 1,381 |
| CKI_2 | CKI | 10x Genomics Chromium | Single Cell 3' v3 | 13,370 | 2,888 | 1,002 |
| CKI_3 | CKI | 10x Genomics Chromium | Single Cell 3' v3 | 16,785 | 2,094 | 810 |
| PTX_1 | PTX | 10x Genomics Chromium | Single Cell 3' v3 | 16,273 | 2,962 | 648 |
| PTX_2 | PTX | 10x Genomics Chromium | Single Cell 3' v3 | 16,565 | 4,096 | 1,279 |
| PTX_3 | PTX | 10x Genomics Chromium | Single Cell 3' v3 | 13,125 | 4,667 | 1,506 |
| PTX+CKI_1 | PTX+CKI | 10x Genomics Chromium | Single Cell 3' v3 | 7,957 | 2,561 | 895 |
| PTX+CKI_2 | PTX+CKI | 10x Genomics Chromium | Single Cell 3' v3 | 13,022 | 2,495 | 896 |
| PTX+CKI_3 | PTX+CKI | 10x Genomics Chromium | Single Cell 3' v3 | 11,878 | 2,996 | 1,018 |
