# Supplementary material for: Single-cell RNA-sequencing uncovers compound kushen injection synergistically improves the efficacy of chemotherapy by modulating the tumor environment of breast cancer
Source: Front Immunol. 2022 Oct 31;13:965342. doi: 10.3389/fimmu.2022.965342 (PMC9660330; doi:10.3389/fimmu.2022.965342)
Supplement: Supplementary file 10 [file Table_3.docx]

**Supplementary Table 3. Metric information for the samples** **subjected to scRNA-seq (after quality control).**

| **Sample ID** | **Group** | **Number of cells** | **Median UMIs per cell** | **Median genes per cell** |
| --- | --- | --- | --- | --- |
| Saline_1 | Saline | 8,364 | 9,364 | 9,364 |
| Saline_2 | Saline | 15,864 | 6,170 | 6,170 |
| Saline_3 | Saline | 10,722 | 8,074 | 8,074 |
| CKI_1 | CKI | 7,545 | 7,661 | 7,661 |
| CKI_2 | CKI | 8,563 | 4,745 | 4,745 |
| CKI_3 | CKI | 9,570 | 3,788 | 3,788 |
| PTX_1 | PTX | 6,771 | 6,584 | 6,584 |
| PTX_2 | PTX | 9,258 | 8,572 | 8,572 |
| PTX_3 | PTX | 7,473 | 9,728 | 9,728 |
| PTX+CKI_1 | PTX+CKI | 4,726 | 3,988 | 3,988 |
| PTX+CKI_2 | PTX+CKI | 8,027 | 4,207 | 4,207 |
| PTX+CKI_3 | PTX+CKI | 8,057 | 4,493 | 4,493 |
